# The centromere comes into focus: from CENP-A nucleosomes to kinetochore connections with the spindle

**DOI:** 10.1098/rsob.200051

**Published:** 2020-06-10

**Authors:** Kathryn Kixmoeller, Praveen Kumar Allu, Ben E. Black

**Affiliations:** 1 Department of Biochemistry and Biophysics, Perelman School of Medicine, University of Pennsylvania, Philadelphia, PA 19104, USA; 2 Penn Center for Genome Integrity, Perelman School of Medicine, University of Pennsylvania, Philadelphia, PA 19104, USA; 3 Epigenetics Institute, Perelman School of Medicine, University of Pennsylvania, Philadelphia, PA 19104, USA; 4 Graduate Program in Biochemistry and Molecular Biophysics, Perelman School of Medicine, University of Pennsylvania, Philadelphia, PA 19104, USA

**Keywords:** centromere, kinetochore, mitosis, chromatin, epigenetics, nucleosome

## Abstract

Eukaryotic chromosome segregation relies upon specific connections from DNA to the microtubule-based spindle that forms at cell division. The chromosomal locus that directs this process is the centromere, where a structure called the kinetochore forms upon entry into mitosis. Recent crystallography and single-particle electron microscopy have provided unprecedented high-resolution views of the molecular complexes involved in this process. The centromere is epigenetically specified by nucleosomes harbouring a histone H3 variant, CENP-A, and we review recent progress on how it differentiates centromeric chromatin from the rest of the chromosome, the biochemical pathway that mediates its assembly and how two non-histone components of the centromere specifically recognize CENP-A nucleosomes. The core centromeric nucleosome complex (CCNC) is required to recruit a 16-subunit complex termed the constitutive centromere associated network (CCAN), and we highlight recent structures reported of the budding yeast CCAN. Finally, the structures of multiple modular sub-complexes of the kinetochore have been solved at near-atomic resolution, providing insight into how connections are made to the CCAN on one end and to the spindle microtubules on the other. One can now build molecular models from the DNA through to the physical connections to microtubules.

## 1. Introduction

Ensuring the faithful propagation of genetic information across generations is one of the most fundamental problems of cell biology. Each chromosome must be properly replicated and segregated during every cell cycle [1]. At mitosis, each pair of sister chromatids must align along the metaphase plate and form proper attachments to spindle microtubules such that the sister chromatids segregate towards opposite poles, leaving one chromatid in each resultant daughter cell [2]. The crucial connection to spindle microtubules occurs at a region of the chromosome called the centromere and is mediated by a large protein assembly called the kinetochore [3–6]. The kinetochore assembles at the centromere and spans the distance between centromeric chromatin and spindle microtubules. The kinetochore serves many functions including ensuring proper bi-orientation of sister chromatids (in mitosis) or bivalents (in meiosis), stabilizing kinetochore–microtubule attachments, and preventing incorrect attachments that could lead to erroneous chromosome segregation [2,7].

**Figure 1.**
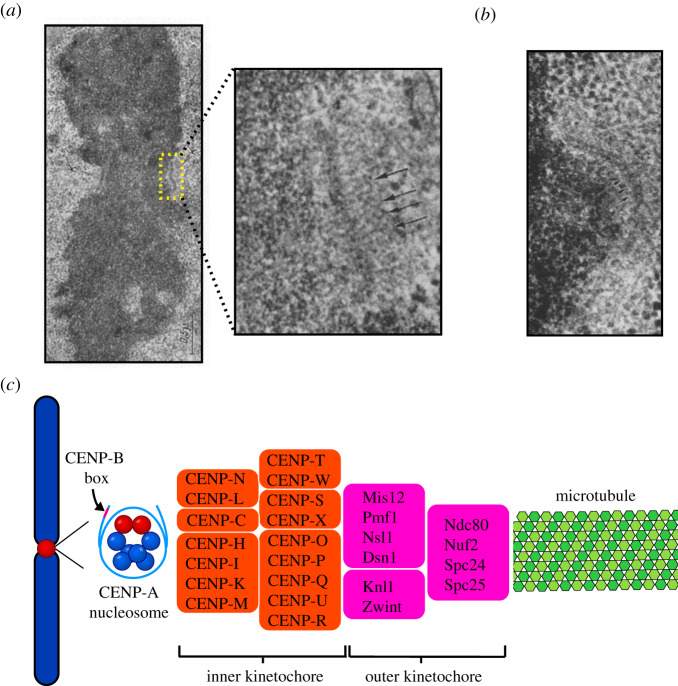
Structural organization of the centromere and kinetochore. (*a*) Electron micrograph of a mitotic chromosome with paired kinetochores on either side of the primary constriction (centromere), prior to microtubule attachments [3]. The inset shows a higher-magnification electron micrograph, which further reveals the trilaminar structure of the kinetochore, showing fibrous elements on either side of the dense central kinetochore plate. Arrows indicate kinetochore fibrils extending out from centromere. Permission to reproduce was sought from the author, Dr William Brinkley, but no response was received. (*b*) This micrograph of the centromere–kinetochore region shows a crescent-shaped kinetochore and the fibrous corona extending outwards from it. (*c*) A schematic of the DNA-microtubule interface, from the CENP-A nucleosomes found in centromeric DNA, through the many subunits of the kinetochore complex, and finally to the microtubule.

The first identification of kinetochore proteins occurred when sera obtained from patients with the autoimmune disease scleroderma were found to contain autoantibodies against the centromere region of chromosomes [8]. The targets of these autoantibodies were three centromeric proteins, named CENP (*CEN*tromeric *P*rotein)-A, CENP-B and CENP-C [9]. Early electron microscopy showed the kinetochore to have a trilaminar structure with a fibrous corona, apparent only when not bound to microtubules, that expands into a crescent shape prior to microtubule binding (figure 1*a*,*b*) [3,10,11]. There has been some debate as to whether this trilaminar structure was due to fixation techniques, and other imaging approaches find the kinetochore to be a fibrous mesh [11–13]. There is broad agreement, though, that the kinetochore provides attachment sites for microtubules that are substantially separated in space from the DNA surface.

In human cells, the foundation of the kinetochore is centromeric chromatin, which contains unusual nucleosomes where CENP-A takes the place of histone H3 [6,14,15]. CENP-B is a DNA-binding protein that interacts with centromeric DNA sequences [16–18]. Moving out from chromatin towards the microtubules, the next part of the kinetochore assembly is the inner kinetochore, which contains many CENP proteins organized in various subcomplexes including CENP-C, CENP-LN, CENP-HIKM, CENP-OPQUR and CENP-TWSX, together termed the constitutive centromere associated network (CCAN) (figure 1*c*) [19–31]. Building on top of the inner kinetochore is the outer kinetochore, containing the Ndc80, Knl1 and Mis12 complexes, which mediate connections to spindle microtubules (figure 1*c*) [31–42]. Our review addresses structural and biophysical features of the CENP-A nucleosome, the 16-subunit CCAN, and the manner in which these complexes form the basis for microtubule binding via the outer kinetochore and other microtubule-associated proteins. Many other proteins that help direct chromosome segregation, including the inner centromeric chromosome passenger complex (CPC), components involved in sister chromatid cohesion and the components of the spindle assembly checkpoint (SAC), will not be discussed here but have been reviewed at length elsewhere [2,32,43–46]. Rather than comprehensively cover all kinetochore components, we outline the central elements of the centromere-to-microtubule connection. Recent advances in structural studies of the kinetochore have given us higher-resolution structures of individual kinetochore components. They provide new details about how they interact with each other, with centromeric DNA and with spindle microtubules. Here, we review this exciting progress and emphasize areas where the findings can be synthesized into emerging models for the mechanisms underlying chromosome segregation.

## 2. Definition of the centromere

Considering the fundamental nature of chromosome segregation, the chromosomal attachment site for binding microtubules exhibits surprisingly large variation across eukaryotes. In many heavily studied systems (budding yeast, fission yeast, fruit fly, mouse, human and others), chromosomes are monocentric, with a single centromere locus where the kinetochore forms and microtubules bind. Many others (worms, some insects, some plants and others) have holocentric chromosomes in which microtubules attach along the full length of the chromosome [47]. Our focus will be on monocentric chromosomes. In many species, centromeres are made up of repetitive DNA sequences [48–53]. The very small, so-called ‘point’ centromeres in the budding yeast *S. cerevisiae* are defined by a specific 125 bp centromere sequence [49,50]. However, in diverse eukaryotes, the centromere is defined epigenetically by the presence of nucleosomes containing CENP-A in place of conventional histone H3 [54]. In many cases, including humans, the location of the centromere is coincident with large stretches of highly repetitive DNA, where the smallest repeating unit is roughly the size of a nucleosome. In humans, for instance, the repetitive DNA is called α-satellite and the smallest repeating unit is 171 bp [51–53].

**Figure 2.**
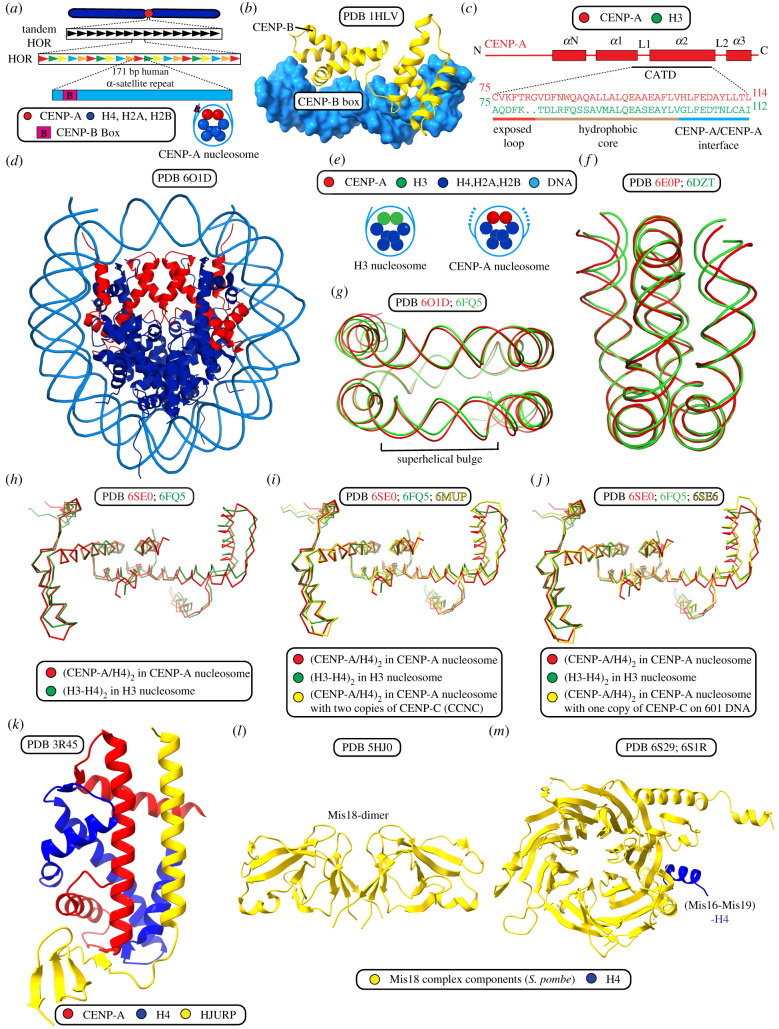
Structure and assembly of CENP-A chromatin. (*a*) Human centromeres typically are located within 0.5–5 Mb of α-satellite DNA arranged in large higher-order repeats (HOR) where the smallest repeating unit is 171 bp. The CENP-B box is located within the human 171 bp α-satellite centromeric repeat monomer, mostly outside of the CENP-A nucleosome DNA entry/exit site. (*b*) The structure of the human CENP-B N-terminal domain as bound to CENP-B box DNA. (*c*) Alignment of the CENP-A targeting domain (CATD) with the corresponding region of canonical histone H3. The CENP-A targeting domain (CATD) provides a distinct surface and features to centromeric CENP-A nucleosomes when compared with canonical H3 nucleosomes. The CATD provides three crucial characteristics: (1) CENP-A/CENP-A interface rotation; (2) strong interactions at the CENP-A/H4 interface due to hydrophobic stitch residues; (3) a protruding loop L1, which gives CCAN specificity) that combine to make the centromeric CENP-A nucleosome distinct. (*d*) Structure of a CENP-A nucleosome assembled on 145 bp human α-satellite DNA. (*e*) Schematic of DNA entry/exit dynamics. CENP-A nucleosomes show precise positioning on 171 bp α-satellite repeat and show flexibility in the terminal DNA predicted by access to MNase digestion. H3 nucleosomes lack these features when assembled on the same repeats. These features are important for the assembly of kinetochore proteins and are specific for CENP-A nucleosomes due to shortened α-1 helix in CENP-A. (*f*) DNA flexibility within the CENP-A nucleosome impacts the path of terminal DNA in all available cryo-EM and X-ray crystal CENP-A nucleosome structures relative to H3 nucleosome structures. The differences in the paths of DNA bound to CENP-A (red) and H3 (green) nucleosomes can be observed by alignment of the nucleosome cryo-EM structures using the (CENP-A/H4)_2_ and (H3/H4)_2_ dimers, as shown here. (*g*) A distinct feature of CENP-A nucleosome structures assembled on α-satellite DNA is the presence of a superhelical bulge, which is absent from H3 nucleosome structures and from CENP-A nucleosome structures assembled on 601 DNA or palindromic α-satellite DNA sequences. The superhelical bulge in the path of the nucleosomal DNA can be observed here by the alignment of CENP-A nucleosomes assembled on α-satellite DNA (red) with H3 nucleosomes assembled on 601 DNA (green) using the CENP-A/H4 dimer and H3/H4 dimer. The superhelical bulge of CENP-A nucleosomes provides a surface for accurate assembly of CCAN proteins. (*h–j*) Illustrations of the overall impact of intrinsic features of CENP-A and DNA sequence on nucleosome structure by alignment of the CENP-A/H4 dimer to the H3/H4 dimer or the CENP-A/H4 dimer, in the presence or absence of CENP-C from available structures. (*h*) CENP-A (red) and H3 (green) nucleosomes on 601 DNA. (*i*) CENP-A (red) and H3 (green) nucleosome on 601 DNA and CENP-A (yellow) nucleosome with CENP-C on α-satellite DNA. (*j*) CENP-A (red), H3 (green) nucleosome and CENP-A with CENP-C nucleosome (yellow) on 601 DNA. (*k*) The structure of the human CENP-A/H4 dimer in complex with its chaperone, HJURP. (*l*) Crystal structure of the CENP-A nucleosome assembly regulator, *S. pombe* Mis18. The structure includes its N-terminal Yippee-like domain, which is known to act as centromere targeting domain and contains a cradle-shaped pocket which binds DNA and is required for Mis18 functions. (*m*) Structure of the CENP-A nucleosome assembly regulatory complex member, *S. pombe* Mis16 with histone H4.

The human regional centromere is made up of a core of homogeneous ordered repeats and CENP-A nucleosomes flanked by outer regions of heterochromatin containing less organized repeats [55]. α-satellite repeats are organized in a higher-order repeat pattern [6,51–53]. α-satellite repeats are not required for specifying the centromere location [56]. They may participate in the formation and stability of pericentromeric heterochromatin, which forms the outer boundary of the centromere [57]. The CENP-B box (figure 2*a*) is a 17 bp motif that exists in a subset of α-satellite monomer repeats. CENP-B binds to this sequence (figure 2*b*; table 1) and contributes to centromere function [9,17,58–60]. The CENP-B box and CENP-B protein are absent from neocentromeres as well as from normal Y chromosomes [17,56]. Furthermore, deletion of CENP-B does not affect viability in mice [61–63]. However, the human Y chromosome, which lacks CENP-B, mis-segregates at a higher rate than other chromosomes [59]. The deletion of CENP-B also magnifies experimental insults to other centromere components [59]. CENP-B has a role in centromere establishment, since first-generation human artificial chromosomes (HACs) require CENP-B protein and a high density of CENP-B boxes on the α-satellite DNA HAC templates [55,64,65]. However, this function of α-satellite DNA and CENP-B can be completely bypassed when CENP-A nucleosomes are initially assembled on the HAC template upon its introduction into cells [66].

**Table 1.** Table of PDB structures. Colour coding to match the figures: yellow, CENP-A nucleosome assembly machinery; blue, structures related to the CENP-A nucleosome and its binding proteins; orange, CCAN components; magenta, outer kinetochore components.

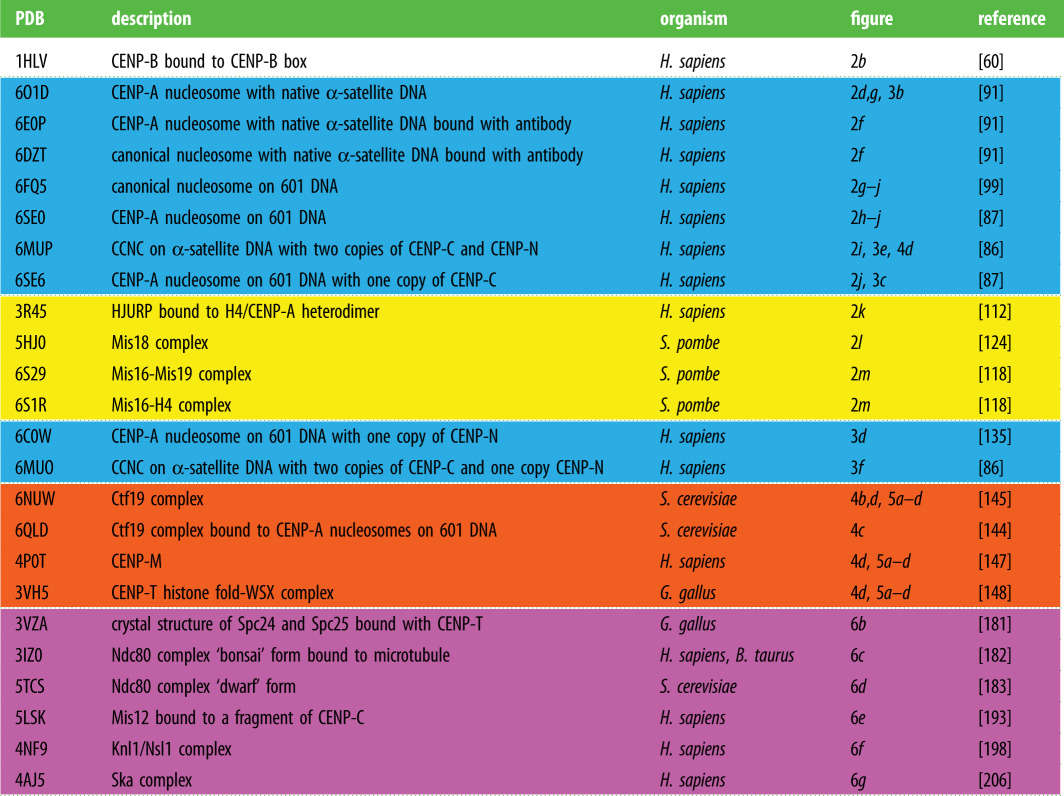

Beyond the presence of repetitive sequences at the centromere, there is also evidence that transcription of centromeric DNA, and the transcripts themselves, are involved in the loading of CENP-A nucleosomes at the centromere and the stabilization of kinetochore components [67–69]. CENP-A also carries many unique post-translational modifications that are probably involved in the epigenetic definition of the centromere. These post-translational modifications have been implicated in many processes central to the centromere including CENP-A incorporation and recruitment of inner kinetochore proteins (reviewed in [70]).

The centromere forms the foundation on which the kinetochore is built. It is the foundation for correct attachments of chromosomes to spindle microtubules and their proper segregation during mitosis or meiosis. For these reasons, the specification of the centromere and its propagation through generations is essential for genetic inheritance, and certainly for cell and organismal viability as well.

## 3. CENP-A nucleosomes

CENP-A nucleosomes form the foundation of the kinetochore assembly and are required, either directly or indirectly, for the localization of all known kinetochore components [71,72]. CENP-A is a variant of histone H3 and exists in chromatin as part of nucleosomes with conventional histones H2A, H2B and H4 [14,15,73,74]. CENP-A nucleosomes are interspersed with canonical H3 nucleosomes at the centromere, and the chromatin must fold in such a way as to expose CENP-A for kinetochore assembly [13,75]. Multiple models have been put forward to explain how this folding occurs in order to expose CENP-A nucleosomes on the surface of the mitotic chromosome [13,76] (reviewed in [6]).

The histone fold domain of CENP-A shares 62% sequence identity with that of histone H3, but its N-terminal ‘histone tail’ shares a basic character but no sequence identity with that of H3 (a large number of Lys residues in H3 are replaced with a large number of Arg residues in CENP-A) [74]. Within the histone fold, the CENP-A targeting domain (CATD) is composed of the first loop (L1) and second α­helix (α2), and is both necessary and sufficient for CENP-A targeting to the centromere (figure 2*c*) [77–80]. The CATD also houses the recognition site for the CCAN component CENP-N [81,82]. CENP-C, another CCAN component, recognizes a 6 a.a. sequence at the extreme C-terminal region of CENP-A [81]. The CATD also appears to support CENP-C recognition in *Xenopus* extracts and in early steps of mammalian centromere formation [83,84], although structural studies [85–87] would suggest this is likely to be an indirect effect through other physical properties of the CENP-A nucleosome imparted by the CATD [88].

CENP-A is incorporated into a nucleosome along with canonical histones H4, H2A and H2B with a histone stoichiometry similar to canonical nucleosomes (figure 2*d*) [88–91] (note that this was controversial, at least in some species [88,90,92–95], but the preponderance of data suggests that the major form in mammals—and probably in many other eukaryotes—is an octamer; reviewed in [54]), but the CENP-A nucleosome has some important physical differences. Arrays of CENP-A nucleosomes more readily form compact structures than their counterparts containing conventional histone H3 [96]. On the individual nucleosome level, however, they have looser connections with the DNA at the nucleosome ‘entry and exit’ sites relative to canonical nucleosomes, made even looser when CENP-C is engaged (figure 2*e*,*f*) [88,89,96–98]. In addition to differences in terminal DNA paths, CENP-A nucleosomes assembled on natural α-satellite DNA also have a distinctive superhelical bulge near superhelical location -3 to -5 (figure 2*g*) [86,91,99]. The α-satellite DNA sequence directs local and global changes in the structure of CENP-A nucleosomes that provide a foundation for CCAN assembly. Within the CENP-A nucleosome, CENP-A forms a more rigid contact point with histone H4 relative to the same point between H3 and H4 in canonical nucleosomes [79,80,88]. CENP-A also directs a steady-state nucleosome conformation in solution where the CENP-A/CENP-A’ interface is rotated relative to the corresponding H3/H3′ interface in canonical nucleosomes (figure 2*h*–*j*) [86–88,91,97,99,100]. Future work is needed to investigate how these features impact the assembly of higher-order complexes at the centromere (i.e. bound to the mammalian CCAN and with natural centromere DNA; more on this below).

The location of centromeres on chromosomes must be conserved across cell generations, so there must exist some mechanism to ensure that CENP-A is maintained at centromeric chromatin during mitosis. Indeed, the level of CENP-A at centromeres is stable across numerous cell divisions [101,102]. CENP-A is partitioned between sister chromatids when DNA is replicated in S-phase [101,103], meaning that CENP-A levels at the centromere are counter-intuitively half-maximal at the crucial cell cycle stage when kinetochores are formed in mitosis. New incorporation of CENP-A happens in G1, after mitotic exit, whereas new H3 is incorporated during DNA replication [101,104,105]. This means that nascent CENP-A (in complex with its binding partner histone H4) must displace H3/H4 when new CENP-A nucleosomes are incorporated in G1 [105].

HJURP is a histone chaperone specific for CENP-A/H4 that is critical for nascent CENP-A nucleosome incorporation in G1 [106,107] and for re-incorporation behind the replication fork [108]. HJURP localizes to the centromere only during G1, and its CENP-A binding domain specifically recognizes and binds to the CATD [106,107,109]. HJURP is also sufficient for incorporation of CENP-A nucleosomes at an ectopic site such as in the formation of HACs [66,110,111]. A high-resolution structure of HJURP in complex with a CENP-A/H4 heterodimer (figure 2*k*) [112] revealed that the C-terminal β-sheet domain of HJURP occludes a major portion of the DNA binding surface of CENP-A/H4. This structure also revealed that, beyond the CATD, Ser68 on the α1-helix of CENP-A also contributes to the binding surface with HJURP [112]. HJURP is restricted from engaging with centromeres and depositing nascent CENP-A protein until cell cycle-dependent restrictions are relieved [113]. HJURP is directly recruited by the Mis18 complex comprosed of MIS18α, MIS18β and M18BP1 (also known as KNL2) (figure 2*l*) [114–118]. The fission yeast *S. pombe* lacks M18BP1, and in this species Mis18 instead forms an analogous complex with Mis16, Mis19 and Mis20 (figure 2*m*) [119–122]. M18BP1 also interacts with CENP-C, which may help to explain how it is targeted to centromeric chromatin and regulates CENP-A deposition [83,123]. A high-resolution structure from the fission yeast *S. pombe* revealed the structure of Mis18 in that species (figure 2*l*) [124]. Interestingly, in the fruit fly, *D. melanogaster*, the HJURP counterpart, Cal1, binds CENP-A by enveloping the CENP-A/H4 heterodimer [125]. Cal1 also guides the deposition of CENP-A at the centromere and directly binds CENP-C, thereby combining the roles of HJURP and the Mis18 complex [125–128]. Understanding exactly how these proteins and others target, deposit and maintain CENP-A at the centromere represents an important challenge for the field.

## 4. Recognition of CENP-A nucleosomes by CENP-C and CENP-N

**Figure 3.**
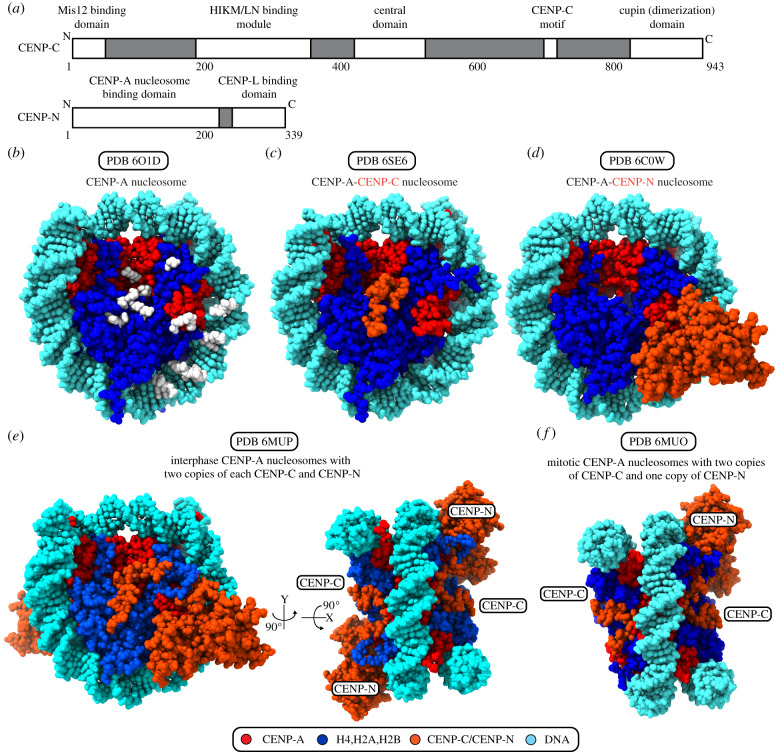
The CCNC forms the foundation of the kinetochore. (*a*) Domain coordinates for human CENP-C and CENP-N. (*b*) The CENP-A nucleosome structure is shown facing the surface of the histone octamer, with the dyad at top. White positions highlight the points on the protein and DNA surface where CCNC components have direct contacts. (*c*) The CENP-A nucleosome assembled with Widom 601 DNA and bound with the central domain of CENP-C. (*d*) The CENP-A nucleosomes assembled with Widom 601 DNA and bound with CENP-N. (*e*) The CENP-A nucleosome assembled with human α-satellite DNA and bound with two copies of CENP-C and CENP-N, proposed to be the interphase form of the CCNC. (*f*) The CENP-A nucleosome assembled with human α-satellite DNA and bound with two copies of CENP-C and one copy of CENP-N, proposed to be the mitotic form of the CCNC.

Surface features on the core of the CENP-A nucleosome distinguish it from canonical nucleosomes containing conventional histone H3 so that it can be recognized by CCAN subunits CENP-C and CENP-N (figure 3) [91].

CENP-C is the ‘keystone’ of the inner kinetochore. It is an elongated protein with binding sites for several centromere and kinetochore components, serving to assemble and organize components of the CCAN [9,26,31,81,129,130]. In mammals, CENP-C has two nucleosome binding domains, the central domain and the CENP-C motif (figure 3*a*) [85]. The CENP-C motif is conserved from budding yeast, and in some species this is the only nucleosome-binding domain of CENP-C. The central domain is conserved only within mammals and has an especially high specificity for CENP-A over H3 compared with the less specific CENP-C motif [85], although recent findings with chicken CENP-C indicate that CDK-mediated phosphorylation of the CENP-C motif can enhance CENP-A nucleosome binding [131]. In humans, the central domain directs the structural transition in the CENP-A nucleosome that occurs upon CENP-C binding and stabilizes CENP-A nucleosomes [97,100,132], an especially important CENP-C activity during DNA replication [103]. In budding yeast, CENP-C^Mif2p^ interacts not only with the histone core of the CENP-A nucleosome but also with the especially AT-rich budding yeast centromere DNA through a continuous DNA and histone-binding surface [133].

The majority of CENP-C is thought to be elongated and largely disordered, so we lack a complete structural model. One globular domain is known to exist: the C-terminal portion of CENP-C, which contains a cupin domain that facilitates homodimerization (figure 3*a*). The CENP-C cupin domain consists of a nine-strand jelly roll folded architecture [134]. This central element of the CENP-C cupin domain is present in budding yeast as well as in organisms with regional centromeres, but the cupin domain of organisms with regional centromeres also contains additional structural features that stabilize its structure and contribute to homodimerization [134].

Along with CENP-C, CENP-N also directly interacts with CENP-A nucleosomes (figure 3*c*–*f*) [81,82]. Together, CENP-A, CENP-C and CENP-N form the core centromeric nucleosome complex (CCNC) [86,132]. Structures are available for CCNC components both alone and in various combinations (figure 3*c*–*f*) [86,87,91,134–137]. The N-terminal portion of CENP-N binds to the CATD of CENP-A (figure 3*a*) [82,132]. This interaction fastens CENP-A to nucleosomal DNA and stabilizes the CENP-A surface bulge [132], supporting the idea that CENP-N provides additional stability to centromeric chromatin beyond what can be attributed to the central domain of CENP-C. The binding of CENP-N to CENP-A is also stabilized by electrostatic interactions with nucleosomal DNA, an observation that is consistent with the coevolution of CENP-N and CENP-A [135,136]. The N-terminal domain of CENP-N does not interact directly with the central domain of CENP-C, but the N-terminal tail of H4 is sandwiched between the C-terminal end of the CENP-C central domain and two loops extending from the CENP-N N-terminal domain, providing a site of indirect interaction between the two non-histone components of the CCNC [86]. When the CCNC is assembled on its favoured natural DNA sequence (α-satellite DNA), there is a distinctive bulge four turns of DNA from the dyad axis that widens the superhelical pitch, making this structure an outlier compared with other nucleosomal structures (figure 2*g*) [86,89,99,135,138]. This DNA bulge probably accommodates a DNA shift favourable for the binding of the CENP-N N-terminal region, allowing two CENP-N molecules to interact equivalently with the nucleosome, one on each surface. Upon mitotic entry, CENP-C levels are constant per unit of centromeric chromatin, while CENP-N levels drop by half, leading to a model wherein the interphase form of the CCNC contains two copies each CENP-C and CENP-N, while the mitotic form contains two copies of CENP-C but only a single copy of CENP-N (figure 3*e*,*f*) [86].

## 5. The inner kinetochore: CCAN structure

**Figure 4.**
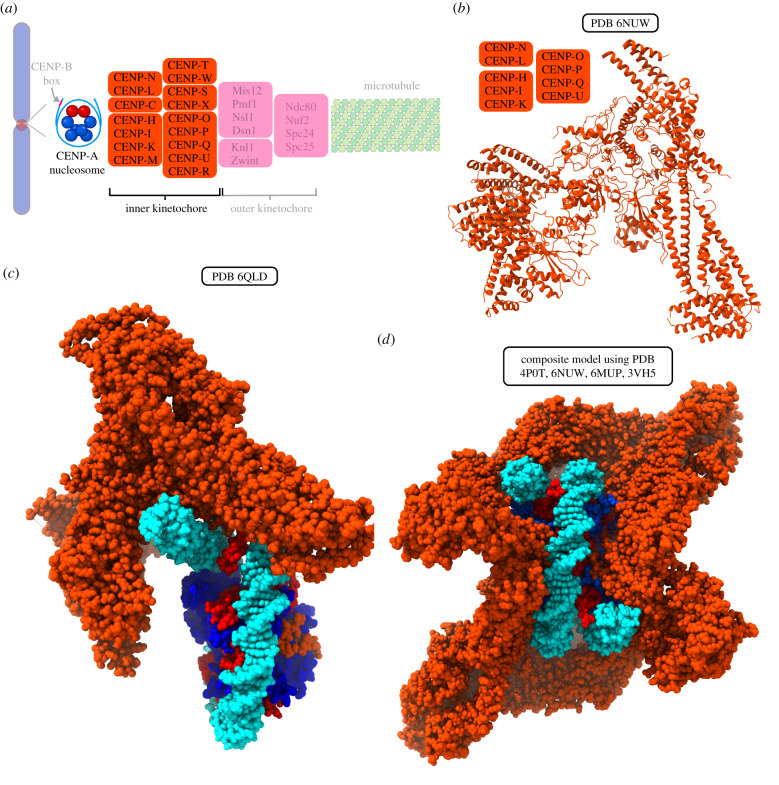
Structure of the yeast CCAN and models of the human CCAN complex bound to CENP-A nucleosome. (*a*) A schematic of the DNA–microtubule interface, with the CENP-A nucleosome and inner kinetochore highlighted. (*b*) Structure of the *S. cerevisiae* Ctf19/CCAN complex containing homologues to human CENP-LN, CENP-HIK and CENP-OPQUR. (*c*) Cryo-EM structure of one copy of *S. cerevisiae* Ctf19 (lacking homologues of CENP-M and CENP-TWSX) interacting with CENP-A nucleosome on Widom 601 DNA. In this structure, CENP-N^Chl14^ does not contact CENP-A or proximal DNA at the sites described in structures of mammalian CENP-N. (*d*) Composite model of CCAN (Ctf19 complex/CENP-M/CENP-TWSX) with the CCNC. Structural alignment was performed by aligning the N-terminal domain of the common subunit, CENP-N.

Biochemical reconstitution and cell-based studies have revealed that, with the exception of CENP-C, the CCAN is composed of four constitutive subcomplexes, each of which is stabilized by reciprocal interactions among its component parts. These subcomplexes are CENP-LN, CENP-HIKM, CENP-TWSX and CENP-OPQUR (figure 4*a*) [22,23,26,139]. As discussed above, CENP-C and CENP-N have both been shown to directly interact with CENP-A and serve as important connections between the inner kinetochore and centromeric chromatin. Most human CCAN proteins have orthologues in the budding yeast (*S. cerevisiae*) Ctf19 complex, which also contains the CBF3 complex that directly binds yeast centromeric DNA and is not present in humans [25,37,140–143].

Major recent progress has been made in understanding the structure of the budding yeast CCAN (also referred to as Ctf19 complex) (figure 4*b*) [144–146]. These structures contain CENP-I^Ctf3^, CENP-H^Mcm16^, CENP-K^Mcm22^, CENP-L^Iml3^, CENP-N^Chl4^, CENP-P^Ctf19^, CENP-O^Mcm21^, CENP-Q^Okp1^, CENP-U^Ame1^, CENP-T^Cnn1^ and CENP-W^Wip1^, as well as Nkp1 and Nkp2, which apparently lack human homologues. Other crystallographic studies have reported the structures of human CENP-M [147] and chicken CENP-TWSX [148]. One of the recent larger structures is of the budding yeast CCAN bound to CENP-A nucleosomes and also includes CENP-C (although most of CENP-C did not produce assignable density except for the small region contacting CENP-A nucleosomes). CENP-S^Mhf1^ and CENP-X^Mhf2^ orthologues were not included, and there are not apparent CENP-M or CENP-R orthologues present in budding yeast [144]. In this structure (figure 4*c*), the orientation of CENP-N places it nowhere near the contact point on CENP-A or the proximal DNA (at SHL 2.5–3.5) described for mammalian CENP-N orthologues (figure 3). Instead, it contacts nucleosomal DNA in an opposite location (SHL 6), indicating that budding yeast CENP-N may not share the direct recognition of CENP-A protein possessed by its mammalian counterparts. The nucleosome in this structure [144] used the strong artificial nucleosome positioning sequence, Widom 601 [149]. While two copies of CENP-C bind to budding yeast CENP-A nucleosomes when assembled on natural centromere DNA [133,144], only a single CENP-C binds when the Widom 601 sequence is used [144]. A parallel situation is present in mammals, where nucleosomes assembled with the natural centromere DNA (human α-satellite) more easily assemble into the CCNC than do those formed on the artificial Widom 601 sequence [86]. This is probably due to the natural flexibility of α-satellite DNA at key locations along the nucleosome surface where CENP-C and CENP-N bind (figure 4*d*). These findings drive home the importance of keeping the choice of DNA sequence in mind when one considers the proposed structural models, since it is possible that artificial sequences may not report accurately on the stoichiometry or local arrangement of CCAN components.

## 6. CENP-C and CENP-LN

Both CENP-C and CENP-N are required to recruit all other CCAN components [26,81–83,85,130,135,136,150]. Thus, when asymmetry arises on an individual CENP-A nucleosome in CENP-C or CENP-N copy number (i.e. in the proposed asymmetric mitotic form of the CCNC) [86], it follows that the surface of the CENP-A nucleosome bound by both CENP-C and CENP-N would recruit the full CCAN capable of nucleating kinetochore formation.

**Figure 5.**
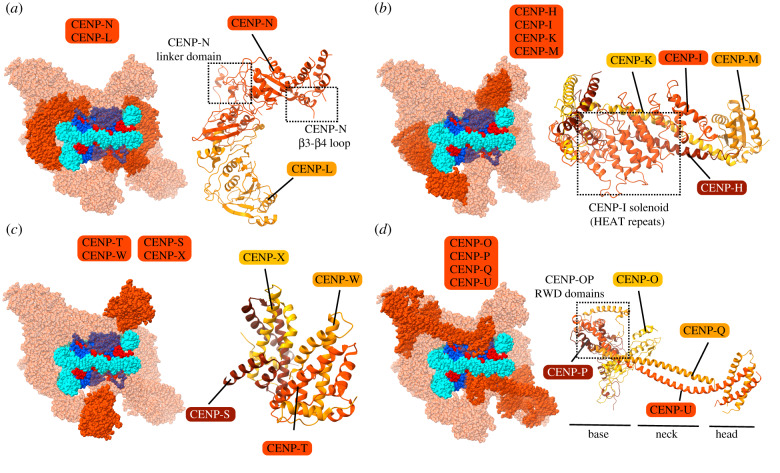
Organization of CCAN sub-complexes on CENP-A nucleosomes. (*a*–*d*) Location of indicated complex within the composite model of CCAN-CCNC complex (left) and ribbon diagram in isolation (right).

One component, CENP-C, acts as a scaffold with modular binding surfaces that can interact with diverse components (CENP-A nucleosomes, CENP-A assembly proteins, CCAN components, self-interactions and outer kinetochore components [26,81,130,132,150,151]), spanning a distance of approximately 100 nm (figure 3*a*) [152]. In contrast, the other component, CENP-N, is only known to interact with CENP-A nucleosomes via its N-terminal globular domain and with its closest binding partner within the CCAN, CENP-L, via its C-terminal domain (figure 3*a*) [82,132,135,136]. Less is known about CENP-L, itself. As a whole, the CENP-LN subcomplex interacts preferentially with CENP-A over H3 [31]. CENP-L^Iml3^-CENP-N^Chl4^ from budding yeast, in the context of the CCAN/Ctf19 complex (figure 5*a*), is thought to be similar in structure to human CENP-LN [145]. The budding yeast structure revealed that the CENP-N^Chl4^ linker domain (which links the N-terminal and C-terminal regions) makes extensive contacts with other CCAN/Ctf19 components, CENP-P^Ctf19^-CENP-O^Mcm21^ [145]. In addition, the β3-β4 loop of CENP-N^Chl4^ extends into the central cavity of the CCAN/Ctf19 complex and contacts the coiled coils of CENP-U^Ame1^-CENP-Q^Okp1^ [145]. Finally, CENP-L^Iml3^ binds directly to the Ctf3 complex (CENP-HIK) providing a link bridging to other components of the CCAN/Ctf19 to the CENP-A nucleosome [145].

## 7. CENP-HIKM

CENP-HIKM is thought to be a single tetrameric unit that is recruited to the kinetochore by CENP-C, and it can bind to CENP-C and CENP-LN within a larger complex: CENP-CHIKMLN [139,145,147]. CENP-HIKM contributes to the CENP-A nucleosome binding affinity of CCNC components, but it alone is not selective for CENP-A nucleosomes relative to canonical nucleosomes containing H3 [139]. CENP-M is a pseudo-GTPase that is required to stabilize CENP-I and is essential to the stability of the CENP-HIKM complex as a whole [147]. Disruption of the interaction between CENP-M and CENP-I leads to defective kinetochore assembly and chromosome alignment [147].

A structure of the budding yeast Ctf3 complex (CENP-HIK) in the context of the CCAN/Ctf19 complex reveals the organization of this subcomplex (figure 5*b*). The C-terminal region of CENP-I^Ctf3^ has multiple HEAT repeats, and a CENP-H^Mcm16^-CENP-K^Mcm22^ coiled-coil motif fits into the concave surface of the CENP-I^Ctf3^ HEAT array [146]. The N-terminal region of CENP-I^Ctf3^ was separately shown to contain HEAT repeats just like the C-terminal region, and the interaction between the C-terminal region of CENP-I^Ctf3^ and CENP-H^Mcm16^-CENP-K^Mcm22^ is mirrored at the other end of the complex [153]. The CENP-H^Mcm16^-CENP-K^Mcm22^ dimer extends through the entirety of this structure. A tight space between the CENP-I^Ctf3^ HEAT array and the CENP-H^Mcm16^-CENP-K^Mcm22^ helices provides a binding site for another CCAN/Ctf19 component, CENP-O^Mcm21^ [145,146]. CENP-M lacks a homologue in yeast and so is absent in the CCAN/Ctf19 complex [144–146]. However, a high-resolution structure of CENP-M in isolation is available and is included in the composite CCAN model (figure 5*b*) [147]. In the CENP-HIKM complex, CENP-M has been proposed to bind within the concave surface of the CENP-I solenoid [147].

## 8. CENP-TWSX

The tetrameric CENP-TWSX complex is made up of the 2-unit CENP-TW and CENP-SX subcomplexes (figure 5*c*) [23,148]. Each component of the CENP-TWSX complex has a histone fold domain [148,154–156]. Unlike CENP-A, CENP-TW turns over frequently throughout the cell cycle, and is incorporated at the centromere in late S and G2 [157]. CENP-T recruitment to the kinetochore involves the CENP-HIKM complex and the N-terminal tail of CENP-A [84,158,159]. Ablating either CENP-TW or CENP-SX leads to the destabilization of the outer kinetochore [22,23]. While in vertebrates CENP-T and CENP-W are both essential proteins, CENP-S and CENP-X are not [22,23]. Nonetheless, in one proposal CENP-TWSX forms a tetrameric structure that organizes DNA in a nucleosome-like complex [148,160]. DNA binding by the CENP-TWSX complex relies on the histone folds of CENP-T and CENP-W and does not appear to exhibit any sequence specificity [148,160]. The CENP-TWSX complex induces positive DNA supercoiling, opposite to the negative supercoiling generated upon canonical nucleosome assembly [148,160]. In the CCAN/Ctf19 complex, CENP-T^Cnn1^ contacts CENP-I^Ctf3^ through an extension of its histone fold motif composed of two additional short helices [145,161]. Furthermore, the orientation of CENP-T^Cnn1^-CENP-W^Wip1^ in the overall CCAN/Ctf19 complex is consistent with binding to DNA [145].

## 9. CENP-OPQUR

The 5-subunit CENP-OPQUR complex (CENP-U is also known as CENP-50) is an important connection between the inner and outer kinetochore. CENP-OPQUR is recruited by the CENP-CHIKMLN complex and binds to a joint interface of CENP-HIKM and CENP-LN [31]. CENP-OP may form a bridge between CENP-CHIKMLN and CENP-QUR. A recent 26-subunit reconstruction of human kinetochore components revealed that CENP-OP and CENP-QU are constitutive subcomplexes whereas CENP-R is stable and soluble in isolation [31]. Similarly, kinetochore recruitment of the units making up the CENP-OPQU complex are interdependent, but CENP-OPQU can localize to the kinetochore in the absence of CENP-R. CENP-R interacts primarily with the CENP-QU subcomplex and does not appear to be required for recovery from spindle damage, unlike the other components of the CENP-OPQUR complex [31]. Although this reconstruction did not yield high-resolution structural information, it showed the CENP-OPQU complex to be bi-lobed with a smaller head and larger base, with the addition of CENP-R adding a protuberance to the neck of the complex and enlargement of its base [31].

Budding yeast do not have a homologue for CENP-R, but the yeast COMA complex is equivalent to human CENP-OPQU (figure 5*d*). The COMA complex is made up of CENP-P^Ctf19^, CENP-Q^Okp1^, CENP-O^Mcm21^ and CENP-U^Ame1^ [37,141]. CENP-U^Ame1^ has been shown to interact with the outer kinetochore MIND complex, analogous to the human Mis12 complex [150], discussed below. The recent high-resolution structures of the yeast CCAN/Ctf19 complex provides more structural detail about this complex [144–146]. CENP-Q^Okp1^ and CENP-U^Ame1^ are structurally intertwined with Nkp1 and Nkp2, which have no clear mammalian orthologues. CENP-Q^Okp1^-CENP-U^Ame1^ and Nkp1/Nkp2 both have helical hairpins in their N-terminal regions, which interact to form a bundle of four helices. The C-terminal regions of the same four proteins form a four-chain helical coil [145,150,162]. CENP-O^Mcm21^ and CENP-P^Ctf19^ have C-terminal RWD domains that interact with the intermediate segments of CENP-Q^Okp1^, CENP-U^Ame1^ and Nkp1 [145,162,163]. CENP-O^Mcm21^ and CENP-P^Ctf19^ were also shown to have N-terminal extensions that are flexible in isolation [145,162]. In the CCAN/Ctf19 complex structure, a helix at the tip of CENP-O^Mcm21^ was shown to interact with the C-terminal region of the CENP-I^Ctf3^ solenoid [145]. Furthermore, deletion of this helix caused CENP-I^Ctf3^ to fail to localize to the kinetochore, suggesting that CENP-O^Mcm21^ may be involved in recruiting the CENP-HIK^Ctf3^ complex to the kinetochore [145]. Nkp1 and Nkp2, crucial elements of this structure in budding yeast, are absent in mammals, as mentioned above, making it unclear to what degree some of the structural information can be extrapolated to the human kinetochore.

The precise role of CENP-OPQUR at the kinetochore remains unclear, but it has been reported to be required for recruiting to the mitotic kinetochore the motor protein, CENP-E, and the mitotic regulatory kinase, PLK1 [164–166]. CENP-QU can bind to microtubules and is capable of sustaining long-term attachments to them *in vitro* [31,167,168]. Relevant to this activity is that the structure of the N-terminal region of CENP-Q is similar to that of the microtubule-binding outer kinetochore protein Ndc80 [31].

## 10. The outer kinetochore

**Figure 6.**
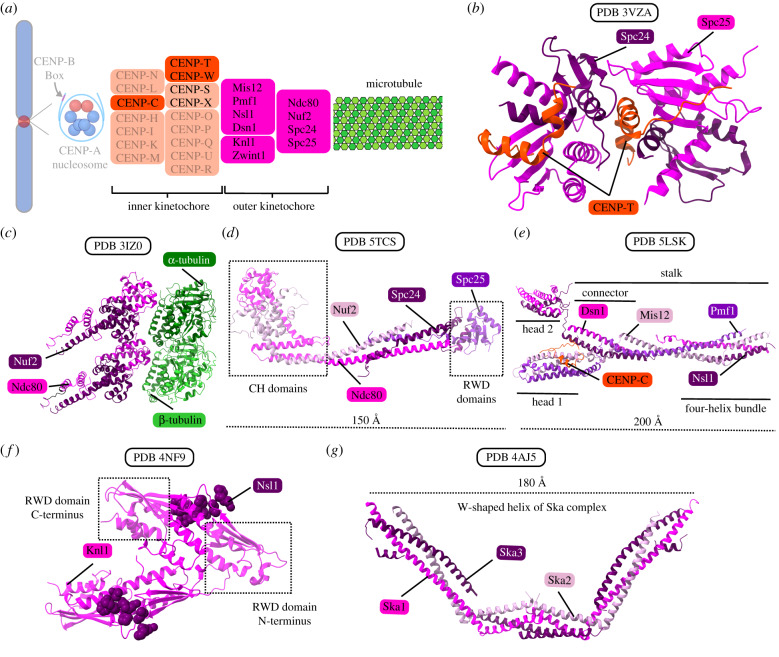
The KMN complex and other microtubule couplers. (*a*) A schematic of the DNA-microtubule interface, highlighting the microtubule, outer kinetochore and components of the inner kinetochore that couple the KMN to CENP-A nucleosomes. (*b*) Crystal structure of chicken Spc24/Spc25 globular domain (part of the Ndc80 complex) bound with CENP-T. This interaction allows up to two Ndc80 complexes to be recruited for each copy of CENP-T. (*c*) Structure of the *S. cerevisiae* ‘bonsai’ chimeric Ndc80 complex bound to tubulin. The ‘bonsai’ structure contains minimal coiled-coil domains, and Ndc80 is fused to Spc25 and Nuf2 to Spc24. The Ndc80 complex binds microtubules via N-terminal calponin homology domains in Ndc80 and Nuf2. (*d*) Structure of the dwarf Ndc80 tetramer, which is shortened with respect to the full complex but maintains the full tetramer junction. (*e*) Crystal structure of the human Mis12 complex bound with a fragment of the CENP-C N-terminal region. The Mis12 complex also interacts with CENP-T, Knl1 and Ndc80, serving as an important interaction hub between the KMN assembly and the inner kinetochore. (*f*) Crystal structure of the C-terminal RWD domains of human Knl1 interacting with Nsl1 residues. Knl1 is the largest structural subunit in the outer kinetochore and provides binding sites for Nsl1 as well as several proteins that interact with the outer kinetochore. (*g*) Crystal structure of the human Ska complex. The Ska complex functions to enhance the microtubule binding ability of the Ndc80 complex.

The outer kinetochore builds on top of the inner kinetochore and is the site of microtubule binding as well as the site of SAC recruitment [7]. The outer kinetochore transduces force from depolymerizing microtubules to ensure proper segregation of sister chromatids towards the spindle poles. The outer kinetochore is primarily made up of the 10-subunit KMN assembly, which contains the Ndc80 complex, Mis12 complex and the Knl1 complex (figure 6*a*) [33,35,39,42,169–171]. There are also numerous proteins beyond the KMN assembly that localize to the outer kinetochore [33,37]. In vertebrates, the complexes of the KMN assembly are not localized to the kinetochore in G1 but instead are recruited in S and G2 phases, with the Ndc80 complex the last to be recruited [172–175]. Thus, the process of preparing the centromere for mitosis is thought of as a stepwise recruitment of outer kinetochore components.

## 11. Ndc80 complex

The four-subunit Ndc80 complex is made up of Ndc80, Nuf2, Spc24 and Spc25, and functions as the primary microtubule receptor on the kinetochore [171,176,177]. The complex is composed of two heterodimers: Ndc80-Nuf2 and Spc24-Spc25 [42,178–180]. The Ndc80-Nuf2 heterodimer has a globular N-terminal region that mediates microtubule binding as well as a C-terminal coiled coil [178–180]. Similarly, the Spc24-Spc25 heterodimer has a C-terminal globular region containing paired RWD domains as well as an N-terminal coiled coil [178–180]. The RWD domains of Spc24 and Spc25 interact with the disordered N-terminal region of CENP-T, allowing up to two Ndc80 complexes to be recruited for each copy of CENP-T (figure 6*b*) [161,174,181]. Each pair of proteins interacts via their coiled-coil domains for dimerization, and then the two heterodimers interact through their dimerized coiled-coil domains to form a tetramer [178–180]. This gives the Ndc80 complex an overall dumbbell-like shape, with a globular domain at each end and coiled coils in between [178–180]. Due to the elongated shape of the Ndc80 complex, no high-resolution structures exist of the full complex in its native form. However, two modified forms provide important structural information that can be extrapolated to understand the structure of the complex as a whole. One such modified structure is the ‘bonsai’ structure, a chimeric Ndc80 complex containing minimal coiled-coil domains, in which Ndc80 is fused to Spc25 and Nuf2 is similarly fused to Spc24 (figure 6*c*) [180,182]. The other is the ‘dwarf’ form, which is shortened with respect to the full complex but maintains the full tetramer junction (figure 6*d*) [183].

The Ndc80 complex binds microtubules via N-terminal calponin homology domains in Ndc80 and Nuf2 (figure 6*c*) [182,184,185]. The basic N-terminal domain of Ndc80 interacts with the acidic E-hook on both α- and β-tubulin [184–186]. Unlike Ndc80, Nuf2 does not contact microtubules directly through its calponin homology domain, but the domain is necessary for high-affinity binding of the Ndc80 complex to microtubules [180,187]. The N-terminal domain of Ndc80 has also been implicated in cooperative binding with other Ndc80 complexes bound along the length of the same protofilament [182,184]. The oligomerization of the Ndc80 complex through the Ndc80 subunit means that a single Ndc80 complex can detach from the microtubule where tubulin is depolymerizing but remain associated with the microtubule through oligomerization [182,184]. This mechanism probably contributes to how the kinetochore can maintain load-bearing attachments to depolymerizing microtubules. It has been shown that, unlike individual Ndc80 complexes in solution, Ndc80 complexes immobilized at high concentration on beads can create load-bearing attachments to depolymerizing microtubules [188–190]. This evidence underscores the importance of cooperative binding in forming stable microtubule attachments. Finally, the Ndc80 complex, and the N-terminal domain of Ndc80 in particular, may also act as a conformation sensor for straight protofilaments [182,191].

Aurora B kinase, a key regulator of kinetochore–microtubule attachments and the catalytic subunit of the CPC, phosphorylates sites on the N-terminal tail of Ndc80, neutralizing its positive charge and thereby decreasing its microtubule binding affinity [182,184]. In this way, phosphorylation by Aurora B kinase serves as a negative regulator of kinetochore–microtubule attachments at the Ndc80 complex. The related Aurora A kinase, which is involved in spindle pole separation in early mitosis, has also been shown to regulate kinetochore–microtubule dynamics in metaphase via phosphorylation of a site in the N-terminal tail of Ndc80 [177].

## 12. Mis12 complex

The four-subunit Mis12 complex contains Mis12, Pmf1, Nsl1 and Dsn1 [192,193]. The budding yeast equivalent of this complex is the MIND (or Mtw1) complex [194,195]. The Mis12 complex interacts with both CENP-C and CENP-T, serving as an important interaction hub between the KMN assembly and the inner kinetochore [196,197]. The complex also provides a binding site for Knl1 and the Ndc80 complex [173,192,198]. As discussed above, CENP-T directly binds up to two Ndc80 complexes. CENP-T also binds Mis12, which then interacts with Ndc80, providing a mechanism by which a third Ndc80 complex can be indirectly recruited by a single CENP-T complex [174]. Since Mis12 also interacts with CENP-C, it has been proposed that two Mis12 complexes could be recruited to by a single CCAN, making it possible for one CCAN to ultimately bind up to four Ndc80 complexes [161,174]. The Mis12 complex is roughly rod-shaped, with high helical content as well as linear motifs (figure 6*e*) [193,194,198].

## 13. Knl1 complex

The Knl1 complex is composed of Knl1 and ZWINT. Knl1 is the largest structural subunit in the outer kinetochore. Its structure is largely intrinsically disordered with the exception of the C-terminal approximately 500 a.a. residues [198]. The C-terminal ordered region contains a coiled coil followed by paired RWD domains, a structure reminiscent of the other kinetochore proteins Scp24, Spc25, CENP-O and CENP-P [162,186,198]. The RWD domains of Knl1 mediate a direct interaction with the stalk of the Mis12 complex (figure 6*f*), and Knl1 also binds many other proteins that associate with the outer kinetochore [198]. ZWINT, the other component of the Knl1 complex, plays a central role in the spindle assembly checkpoint (SAC), an essential pathway that ensures proper chromosome alignments and kinetochore–microtubule attachments before proceeding with mitosis [199–203].

## 14. Connections to microtubules

As discussed above, the Ndc80 complex represents a central component in mediating kinetochore–microtubule interactions. However, several other proteins can make these connections and/or physically support these interactions. Here we will highlight a few key players.

The Ska complex, a trimer of Ska1, Ska2 and Ska3, functions to enhance the microtubule binding ability of the Ndc80 complex [204–206]. The Ska complex has a ‘W’-like shape and is composed of dimers of helical bundles of its subunits (figure 6*g*) [206]. The C-terminal winged-helix domain of Ska1 interacts with tubulin and, unlike the Ndc80 complex, indiscriminately tracks the tips of both polymerizing and depolymerizing microtubules [207–209]. The unstructured C-terminal region of Ska3 also facilitates interaction between the complex and microtubules [207]. The budding yeast equivalent of the Ska complex appears to be the Dam1 complex [210–213], although the two complexes have no structural similarity. Beyond the Ska complex, multiple proteins including dynein, kinesin, CENP-E and CENP-F have been shown to form weak attachments to microtubules in the absence of the Ndc80 complex [214–216], suggesting that they may also be involved in kinetochore–microtubule interactions.

A striking feature of the metazoan regional kinetochore is that it expands into a crescent-like shape prior to end-on microtubule binding by the Ndc80 complex (figure 1*b*) [10,11,217]. This process involves numerous molecules including CENP-E, CENP-F, the dynein/dynactin motor complex and the ROD–ZW10–Zwilch (RZZ) complex [11,214,218,219]. This physical transformation of the kinetochore may serve to increase the likelihood of microtubule capture and may also promote SAC signalling [11]. Once end-on microtubule attachments to the Ndc80 complex are established, the kinetochore retracts from this expanded crescent shape into a smaller plate [11,220]. This retraction of the kinetochore involves the release of the dynein/dynactin and RZZ complexes along with the central SAC complex, halting SAC signalling at that kinetochore [221–227].

Many additional proteins play important roles in forming, maintaining and regulating connections at the kinetochore. These include the SKAP-Astrin complex (which regulates proper kinetochore–microtubule attachments [228–232]), XMAP215 (which is involved in microtubule polymerization and nucleation [233–237]), the CLASPs (which regulate microtubule dynamics [238–241]), MCAK (which is a microtubule depolymerase [242–246]), and more. Discussion of these proteins is beyond the scope of this review, but the kinetochore–microtubule interface has been reviewed elsewhere in depth [247].

## 15. Putting the pieces together—models of the chromatin-to-microtubule connection

**Figure 7.**
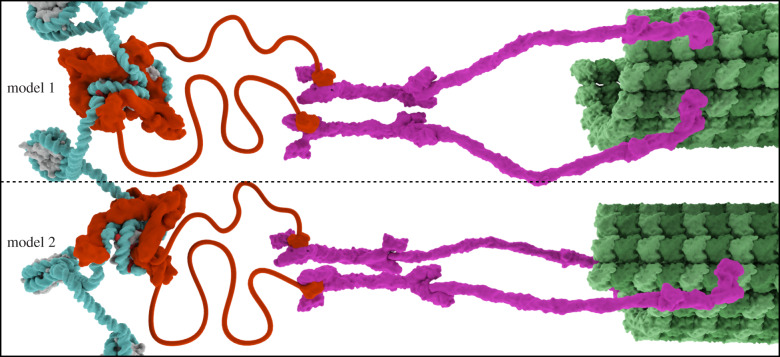
Models of the kinetochore organization on centromeric chromatin. Model 1 has a symmetric arrangement of two copies of the CCAN bound per CENP-A nucleosome, whereas model 2 has an asymmetric arrangement with a single copy of the CCAN per CENP-A nucleosome. Both models contain two copies of CENP-C per CENP-A nucleosome. The proposed connection through CENP-C to neighbouring nucleosomes has been omitted from both models for visual clarity. See text for details about the two models, the data supporting each one and implications for centromeric chromatin structure during mitotic chromosome segregation.

Because of the overall symmetry of the octameric CENP-A nucleosome, harbouring two histone surfaces or ‘faces’ for equivalent recruitment of the recognition subunits of the CCAN (i.e. CENP-C and CENP-N that form the CCNC), the most prominent models for the chromatin connection to microtubules have generally featured a stoichiometry of two CCAN complexes per CENP-A nucleosome (figure 7, model 1) [5]. This has been supported by the sedimentation behaviour of reconstituted particles containing a large subset of recombinant CCAN components [139]. An alternative model has emerged, though, where a single CCAN connects the CENP-A nucleosome to the outer kinetochore (figure 7, model 2) [86,144]. The evidence for the alternate model comes from structural work that strongly implies that the sequence of the underlying DNA can influence stoichiometry and the fidelity of assembly of the CCNC [86,135,136,144] and observations of cell cycle changes in the stoichiometry within the CCNC (i.e. losing one copy of CENP-N at mitotic onset) [86]. Since both CENP-C and CENP-N within the CCNC are required for the assembly of the CCAN, it is likely that both constituents are present on the face of the nucleosome that assembles the CCAN. Analysis of the composition of tagged centromere proteins in mammalian cells have indeed reported that CENP-C is super-stoichiometric to at least one CCAN subunit, CENP-T [248]. Further investigation of the stoichiometry of individual sub-complexes of the CCAN at the onset of mitosis will reveal whether or not there are other mechanisms to adjust its composition at the time of kinetochore assembly.

The two models are primarily presented here to interrogate the implications of the orientation of CENP-A nucleosomes at the interface between the chromosome and the cytosolic surface where microtubules attach. Microtubule attachment is at a perpendicular orientation to the chromosome (typically termed ‘end-on’). The models are not intended to predict differences in the stoichiometry of outer kinetochore components, since there are many ways in which their stoichiometry could be modulated (e.g. expression levels limiting a single connector component or homo-oligomerization of a sub-complex that amplifies some subunits). Further, either model can readily accommodate prior subunit distance measurements made at natural mitotic kinetochores [152,249].

Regarding CENP-A nucleosome orientation, in model 1, the two histone surfaces are equivalently used to recruit an entire CCAN, and the orientation is a symmetric one. One could imagine several orientations of the CENP-A nucleosome, and we illustrate it with the dyad rotated 90° from the microtubule. This would have the entry/exit DNA from the CENP-A nucleosome slightly peeling off the histone octamer and proceeding towards the neighbouring nucleosomes. A dyad position towards the microtubule is more difficult to imagine because this would be the same surface as the exit/entry DNA. Connection to neighbouring nucleosomes, in this case, could be accomplished by a greater amount of DNA unwrapping from the CENP-A nucleosome core, however. In model 2, the histone surfaces are not equivalent. On the surface facing the microtubule, a full CCAN complex is assembled and this nucleates the kinetochore. On the opposite surface, facing the chromosome (i.e. mainly abutting conventional nucleosomes containing histone H3), the full CCAN is absent, but a second copy of CENP-C is present. One potential outcome of this orientation is to use the copy of CENP-C on the chromosome face (and not engaged with the CCAN) to physically connect CENP-A nucleosomes to neighbouring nucleosomes. These connections could help rigidify CENP-A chromatin under spindle pulling forces, since CENP-A chromatin does not substantially deform under these forces, whereas the intervening chromatin at the inner centromere is substantially elongated at mitosis [250].

Another question that must be resolved is how well conserved subunit stoichiometry and arrangement at the centromere are between species. On one hand, one might argue that since the process of chromosome segregation is so fundamental to eukaryotic life that there must be conservation of structure even among diverse species (e.g. budding yeast and humans). From this viewpoint, the surprising arrangement of CENP-L^Iml3^-CENP-N^Chl4^ away from the histone surface of CENP-A in the recent CENP-A nucleosome CCAN/Ctf19 structure (figure 4*c*) [144] (in the face of strong evidence from metazoan systems that CENP-N binds elsewhere; figure 3*d*–*f*) might lead one to question the validity of the structure, itself. On the other hand, we know that there is rampant evolutionary innovation at the centromere [251]. One example of this is the DNA sequence defined elements in budding yeast that recruit CBF1 and CBF3 complexes [252,253]. Another is the recurrent complete loss of CENP-A nucleosomes (and many other CCAN components) in some insect lineages [254]. From that point of view, an innovation like repositioning CENP-L^Iml3^-CENP-N^Chl4^ is not so difficult to imagine.

Regarding the impact of DNA sequence, the experimental findings come primarily from comparing CENP-A nucleosomes assembled with natural centromere DNA to those assembled with a synthetic nucleosome positioning sequence used nearly ubiquitously in chromatin structural biochemistry, the Widom ‘601’ sequence [149], as mentioned above. Cryo-EM analysis of CENP-A nucleosomes assembled with the 601 sequence and bound by CENP-N initially revealed high-resolution structural models with a form of the complex containing a single copy of CENP-N [135,136]. Assembly of CENP-A nucleosomes on α-satellite DNA leads to robust formation of the CCNC with two copies each bound of CENP-C and CENP-N, relative to those assembled with the 601 DNA sequence [86]. This led to single-particle populations yielding high-resolution cryo-EM structures of forms containing two copies of CENP-C and either one or two copies of CENP-N [86]. The AT-rich nature of natural centromeric DNA imparts flexibility that the strong nucleosome positioning 601 sequence lacks, permitting bends in the DNA and a DNA path that creates symmetrical binding sites on each side of the nucleosome. In yeast, centromeric DNA is extremely AT-rich (greater than 85%), and, as mentioned above, this has been reported to affect the stoichiometry of the other CCNC component, CENP-C [144]. Only a single copy of CENP-C^Mif2^ binds CENP-A nucleosomes assembled on 601 DNA [144], whereas two copies of CENP-C^Mif2^ bind CENP-A assembled on yeast CEN DNA [133,144]. It is tempting to speculate that some natural DNA sequences could be refractory to centromere formation because, like the artificial 601 sequence, they do not accommodate the assembly of the CCAN starting with CENP-C and/or CENP-N. These effects certainly not all-or-none, however, since solution biochemistry experiments supporting model 1 (figure 7) described above were performed using the 601 sequence [139], and the cryo-EM experiment on 601 DNA described above also identified a minority of particles containing two copies of CENP-N, a fraction which increased in the presence of excess CENP-N [136]. Analysis of the high-resolution 601 DNA CENP-A nucleosome structures reveals marked asymmetry of the two histone surfaces of the nucleosome [86]. It is most likely that the assembly and stoichiometry of the CCAN is affected by a variety of factors, including DNA sequence, but also reconstitution conditions and variation among species. The field must continue to investigate CCAN assembly and structure *in vitro*, but must also meet the challenge of devising compelling ways to test the hypotheses emerging from these studies on natural centromeres in cells.

Regarding the cell cycle regulation, the relative abundance of CENP-N to CENP-C drops at centromeres immediately at the onset of mitosis [86]. Since both CENP-C and CENP-N are required for the recruitment of other CCAN components to CENP-A nucleosomes [26,81–83,85,130,135,136,150], and since CENP-A nucleosomes do not change in number upon mitotic entry [101], this finding suggests that the number of CCAN complexes per unit of centromeric chromatin drops by half prior to formation of the mitotic kinetochore. The model with a single CCAN per CENP-A nucleosome (figure 7, model 2) is built upon the assumption that most individual constitutive centromere components assemble into discrete CCAN complexes bound to the CENP-A nucleosome. The extensive contacts across the majority of CCAN components evident from the recent structural advances (figures 4 and 5) [86,144–146,148] support the notion that the CCAN can exist as a discrete unit. This does not rule out the possibility that CCAN stoichiometry can be modulated in important ways, and indeed there is evidence that binding surfaces with the CCAN can be rearranged in mitosis [131,255].

Future efforts will be required to differentiate between existing models (figure 7) and will also probably lead to discovery of other important ways in which the CCAN is assembled and modulated as it is in the process of nucleating kinetochore formation.

## 16. Outlook

Proper assembly of the kinetochore at the centromere is a fundamental process of cell biology that is crucial for accurate propagation of genetic information through cell and organismal generations. However, in spite of recent landmark achievements in the structural understanding of the centromere and kinetochore, there remains much to be learned about the structure, organization and function of the centromere and kinetochore components that make this process possible. Determining the precise mechanism of the deposition of CENP-A nucleosomes represents an important challenge for the field. Furthermore, the most comprehensive structures for inner kinetochore components come from budding yeast, and although many yeast kinetochore proteins share homology with their human counterparts, structures of centromere complexes from other species, including from metazoan species, will be critical. They will help to develop an understanding of which structural aspects are universal in eukaryotes and which are undergoing innovations that accompany chromosome evolution. As with essentially all macromolecular complexes, connecting structures of purified/reconstituted complexes to the functionally important structures that exist *in vivo* represents a major hurdle, but one that is crucial to pursue, especially since the CCAN undergoes changes depending on cell cycle state. Technological developments continue to allow us to study structures at higher resolution and in more native contexts, promising a period in the coming years of exciting progress to reveal precisely how the kinetochore connects centromeric chromatin to spindle microtubules (table 1).

## Supplementary Material

Reviewer comments

## Data accessibility

This article has no additional data.

## References

[1] Rieder CL, Salmon ED 1998 The vertebrate cell kinetochore and its roles during mitosis. Trends Cell Biol. 8, 310–318. (10.1016/S0962-8924(98)01299-9) 9704407 PMC4774253

[2] Lampson MA, Grishchuk EL 2017 Mechanisms to avoid and correct erroneous kinetochore-microtubule attachments. Biology 6, 1 (10.3390/biology6010001) PMC5371994 28067761

[3] Brinkley BR, Stubblefield E 1966 The fine structure of the kinetochore of a mammalian cell *in vitro*. Chromosoma 19, 28–43. (10.1007/BF00332792) 5912064

[4] Cooke CA, Bazett-Jones DP, Earnshaw WC, Rattner JB 1993 Mapping DNA within the mammalian kinetochore. J. Cell Biol. 120, 1083–1091. (10.1083/jcb.120.5.1083) 7679671 PMC2119735

[5] Musacchio A, Desai A 2017 A molecular view of kinetochore assembly and function. Biology 6, 5 (10.3390/biology6010005) PMC5371998 28125021

[6] Fukagawa T, Earnshaw WC 2014 The centromere: chromatin foundation for the kinetochore machinery. Dev. Cell 30, 496–508. (10.1016/j.devcel.2014.08.016) 25203206 PMC4160344

[7] Cheeseman IM, Desai A 2008 Molecular architecture of the kinetochore–microtubule interface. Nat. Rev. Mol. Cell Biol. 9, 33–46. (10.1038/nrm2310) 18097444

[8] Moroi Y, Peebles C, Fritzler MJ, Steigerwald J, Tan EM 1980 Autoantibody to centromere (kinetochore) in scleroderma sera. Proc. Natl Acad. Sci. USA 77, 1627–1631. (10.1073/pnas.77.3.1627) 6966403 PMC348550

[9] Earnshaw WC, Rothfield N 1985 Identification of a family of human centromere proteins using autoimmune sera from patients with scleroderma. Chromosoma 91, 313–321. (10.1007/BF00328227) 2579778

[10] Rieder CL 1982 The formation, structure, and composition of the mammalian kinetochore and kinetochore fiber. Int. Rev. Cytol. 79, 1–58. (10.1016/S0074-7696(08)61672-1) 6185450

[11] Magidson V, Paul R, Yang N, Ault JG, O'Connell CB, Tikhonenko I, McEwen BF, Mogilner A, Khodjakov A 2015 Adaptive changes in the kinetochore architecture facilitate proper spindle assembly. Nat. Cell Biol. 17, 1134–1144. (10.1038/ncb3223) 26258631 PMC4553083

[12] Dong Y, Vanden Beldt KJ, Meng X, Khodjakov A, McEwen BF 2007 The outer plate in vertebrate kinetochores is a flexible network with multiple microtubule interactions. Nat. Cell Biol. 9, 516–522. (10.1038/ncb1576) 17435749 PMC2895818

[13] Blower MD, Sullivan BA, Karpen GH 2002 Conserved organization of centromeric chromatin in flies and humans. Dev. Cell 2, 319–330. (10.1016/s1534-5807(02)00135-1) 11879637 PMC3192492

[14] Palmer DK, O'Day K, Wener MH, Andrews BS, Margolis RL 1987 A 17-kD centromere protein (CENP-A) copurifies with nucleosome core particles and with histones. J. Cell Biol. 104, 805–815. (10.1083/jcb.104.4.805) 3558482 PMC2114441

[15] Palmer DK, O'Day K, Trong HL, Charbonneau H, Margolis RL 1991 Purification of the centromere-specific protein CENP-A and demonstration that it is a distinctive histone. Proc. Natl Acad. Sci. USA 88, 3734–3738. (10.1073/pnas.88.9.3734) 2023923 PMC51527

[16] Earnshaw WC, Sullivan KF, Machlin PS, Cooke CA, Kaiser DA, Pollard TD, Rothfield NF, Cleveland DW 1987 Molecular cloning of cDNA for CENP-B, the major human centromere autoantigen. J. Cell Biol. 104, 817–829. (10.1083/jcb.104.4.817) 2435739 PMC2114438

[17] Masumoto H, Masukata H, Muro Y, Nozaki N, Okazaki T 1989 A human centromere antigen (CENP-B) interacts with a short specific sequence in alphoid DNA, a human centromeric satellite. J. Cell Biol. 109, 1963–1973. (10.1083/jcb.109.5.1963) 2808515 PMC2115871

[18] Gamba R, Fachinetti D 2020 From evolution to function: two sides of the same CENP-B coin? Exp. Cell Res. 390, 111959 (10.1016/j.yexcr.2020.111959) 32173469

[19] Nishihashi A, Haraguchi T, Hiraoka Y, Ikemura T, Regnier V, Dodson H, Earnshaw WC, Fukagawa T 2002 CENP-I is essential for centromere function in vertebrate cells. Dev. Cell 2, 463–476. (10.1016/s1534-5807(02)00144-2) 11970896

[20] Sugata N, Munekata E, Todokoro K 1999 Characterization of a novel kinetochore protein, CENP-H. J. Biol. Chem. 274, 27 343–27 346. (10.1074/jbc.274.39.27343) 10488063

[21] Foltz DR, Jansen LET, Black BE, Bailey AO, Yates JR, Cleveland DW 2006 The human CENP-A centromeric nucleosome-associated complex. Nat. Cell Biol. 8, 458–469. (10.1038/ncb1397) 16622419

[22] Hori T et al 2008 CCAN makes multiple contacts with centromeric DNA to provide distinct pathways to the outer kinetochore. Cell 135, 1039–1052. (10.1016/j.cell.2008.10.019) 19070575

[23] Amano M, Suzuki A, Hori T, Backer C, Okawa K, Cheeseman IM, Fukagawa T 2009 The CENP-S complex is essential for the stable assembly of outer kinetochore structure. J. Cell Biol. 186, 173–182. (10.1083/jcb.200903100) 19620631 PMC2717651

[24] Joglekar AP, Bloom K, Salmon ED 2009 *In vivo* protein architecture of the eukaryotic kinetochore with nanometer scale accuracy. Curr. Biol. 19, 694–699. (10.1016/j.cub.2009.02.056) 19345105 PMC2832475

[25] Pot I, Measday V, Snydsman B, Cagney G, Fields S, Davis TN, Muller EGD, Hieter P 2003 Chl4p and Iml3p are two new members of the budding yeast outer kinetochore. Mol. Biol. Cell 14, 460–476. (10.1091/mbc.e02-08-0517) 12589047 PMC149985

[26] McKinley KL, Sekulic N, Guo LY, Tsinman T, Black BE, Cheeseman IM 2015 The CENP-L-N complex forms a critical node in an integrated meshwork of interactions at the centromere–kinetochore interface. Mol. Cell 60, 886–898. (10.1016/j.molcel.2015.10.027) 26698661 PMC4690846

[27] Okada M, Cheeseman IM, Hori T, Okawa K, McLeod IX, Yates JR, Desai A, Fukagawa T 2006 The CENP-H-I complex is required for the efficient incorporation of newly synthesized CENP-A into centromeres. Nat. Cell Biol. 8, 446–457. (10.1038/ncb1396) 16622420

[28] Fukagawa T, Pendon C, Morris J, Brown W 1999 CENP-C is necessary but not sufficient to induce formation of a functional centromere. EMBO J. 18, 4196–4209. (10.1093/emboj/18.15.4196) 10428958 PMC1171496

[29] Liu S-T, Hittle JC, Jablonski SA, Campbell MS, Yoda K, Yen TJ 2003 Human CENP-I specifies localization of CENP-F, MAD1 and MAD2 to kinetochores and is essential for mitosis. Nat. Cell Biol. 5, 341–345. (10.1038/ncb953) 12640463

[30] Fukagawa T et al 2001 CENP-H, a constitutive centromere component, is required for centromere targeting of CENP-C in vertebrate cells. EMBO J. 20, 4603–4617. (10.1093/emboj/20.16.4603) 11500386 PMC125570

[31] Pesenti ME et al 2018 Reconstitution of a 26-subunit human kinetochore reveals cooperative microtubule binding by CENP-OPQUR and NDC80. Mol. Cell 71, 923–939.e10. (10.1016/j.molcel.2018.07.038) 30174292 PMC6162344

[32] Foley EA, Kapoor TM 2013 Microtubule attachment and spindle assembly checkpoint signalling at the kinetochore. Nat. Rev. Mol. Cell Biol. 14, 25–37. (10.1038/nrm3494) 23258294 PMC3762224

[33] Cheeseman IM, Chappie JS, Wilson-Kubalek EM, Desai A 2006 The conserved KMN network constitutes the core microtubule-binding site of the kinetochore. Cell 127, 983–997. (10.1016/j.cell.2006.09.039) 17129783

[34] Cheeseman IM, Niessen S, Anderson S, Hyndman F, Yates JR, Oegema K, Desai A 2004 A conserved protein network controls assembly of the outer kinetochore and its ability to sustain tension. Genes Dev. 18, 2255–2268. (10.1101/gad.1234104) 15371340 PMC517519

[35] Desai A, Rybina S, Müller-Reichert T, Shevchenko A, Shevchenko A, Hyman A, Oegema K 2003 KNL-1 directs assembly of the microtubule-binding interface of the kinetochore in *C. elegans*. Genes Dev. 17, 2421–2435. (10.1101/gad.1126303) 14522947 PMC218079

[36] Nekrasov VS, Smith MA, Peak-Chew S, Kilmartin JV 2003 Interactions between centromere complexes in *Saccharomyces cerevisiae*. Mol. Biol. Cell 14, 4931–4946. (10.1091/mbc.E03-06-0419) 14565975 PMC284796

[37] De Wulf P, McAinsh AD, Sorger PK. 2003 Hierarchical assembly of the budding yeast kinetochore from multiple subcomplexes. Genes Dev. 17, 2902–2921. (10.1101/gad.1144403) 14633972 PMC289150

[38] Kline SL, Cheeseman IM, Hori T, Fukagawa T, Desai A 2006 The human Mis12 complex is required for kinetochore assembly and proper chromosome segregation. J. Cell Biol. 173, 9–17. (10.1083/jcb.200509158) 16585270 PMC2063780

[39] Obuse C, Iwasaki O, Kiyomitsu T, Goshima G, Toyoda Y, Yanagida M 2004 A conserved Mis12 centromere complex is linked to heterochromatic HP1 and outer kinetochore protein Zwint-1. Nat. Cell Biol. 6, 1135–1141. (10.1038/ncb1187) 15502821

[40] DeLuca JG, Dong Y, Hergert P, Strauss J, Hickey JM, Salmon ED, McEwen BF 2005 Hec1 and Nuf2 are core components of the kinetochore outer plate essential for organizing microtubule attachment sites. Mol. Biol. Cell 16, 519–531. (10.1091/mbc.e04-09-0852) 15548592 PMC545888

[41] McCleland ML, Kallio MJ, Barrett-Wilt GA, Kestner CA, Shabanowitz J, Hunt DF, Gorbsky GJ, Stukenberg PT 2004 The vertebrate Ndc80 complex contains Spc24 and Spc25 homologs, which are required to establish and maintain kinetochore-microtubule attachment. Curr. Biol. 14, 131–137. (10.1016/j.cub.2003.12.058) 14738735

[42] Wigge PA, Kilmartin JV 2001 The Ndc80p complex from *Saccharomyces cerevisiae* contains conserved centromere components and has a function in chromosome segregation. J. Cell Biol. 152, 349–360. (10.1083/jcb.152.2.349) 11266451 PMC2199619

[43] Kitagawa M, Lee SH 2015 The chromosomal passenger complex (CPC) as a key orchestrator of orderly mitotic exit and cytokinesis. Front. Cell Dev. Biol. 3, 14 (10.3389/fcell.2015.00014) 25798441 PMC4350427

[44] Dou Z, Prifti DK, Gui P, Liu X, Elowe S, Yao X 2019 Recent progress on the localization of the spindle assembly checkpoint machinery to kinetochores. Cells 8, 278 (10.3390/cells8030278) PMC6468716 30909555

[45] Mirkovic M, Oliveira RA 2017 Centromeric cohesin: molecular glue and much more. Prog. Mol. Subcell. Biol. 56, 485–513. (10.1007/978-3-319-58592-5_20) 28840250

[46] Musacchio A 2015 The molecular biology of spindle assembly checkpoint signaling dynamics. Curr. Biol. 25, R1002–R1018. (10.1016/j.cub.2015.08.051) 26485365

[47] Guerra M, Cabral G, Cuacos M, González-García M, González-Sánchez M, Vega J, Puertas MJ 2010 Neocentrics and holokinetics (holocentrics): chromosomes out of the centromeric rules. Cytogenet. Genome Res. 129, 82–96. (10.1159/000314289) 20551611

[48] Pardue ML, Gall JG 1970 Chromosomal localization of mouse satellite DNA. Science 168, 1356–1358. (10.1126/science.168.3937.1356) 5462793

[49] Clarke L 1998 Centromeres: proteins, protein complexes, and repeated domains at centromeres of simple eukaryotes. Curr. Opin. Genet. Dev. 8, 212–218. (10.1016/S0959-437X(98)80143-3) 9610412

[50] Clarke L, Carbon J 1980 Isolation of a yeast centromere and construction of functional small circular chromosomes. Nature 287, 504–509. (10.1038/287504a0) 6999364

[51] Waye JS, Willard HF 1989 Human beta satellite DNA: genomic organization and sequence definition of a class of highly repetitive tandem DNA. Proc. Natl Acad. Sci. USA 86, 6250–6254. (10.1073/pnas.86.16.6250) 2762326 PMC297815

[52] Vissel B, Choo KH 1987 Human alpha satellite DNA-consensus sequence and conserved regions. Nucleic Acids Res. 15, 6751–6752. (10.1093/nar/15.16.6751) 3628014 PMC306152

[53] Manuelidis L 1978 Chromosomal localization of complex and simple repeated human DNAs. Chromosoma 66, 23–32. (10.1007/BF00285813) 639625

[54] Black BE, Cleveland DW 2011 Epigenetic centromere propagation and the nature of CENP-A nucleosomes. Cell 144, 471–479. (10.1016/j.cell.2011.02.002) 21335232 PMC3061232

[55] Schueler MG, Higgins AW, Rudd MK, Gustashaw K, Willard HF 2001 Genomic and genetic definition of a functional human centromere. Science 294, 109–115. (10.1126/science.1065042) 11588252

[56] Voullaire LE, Slater HR, Petrovic V, Choo KH 1993 A functional marker centromere with no detectable alpha-satellite, satellite III, or CENP-B protein: activation of a latent centromere? Am. J. Hum. Genet. 52, 1153–1163. 7684888 PMC1682274

[57] McNulty SM, Sullivan BA 2018 Alpha satellite DNA biology: finding function in the recesses of the genome. Chromosome Res. 26, 115–138. (10.1007/s10577-018-9582-3) 29974361 PMC6121732

[58] Muro Y, Masumoto H, Yoda K, Nozaki N, Ohashi M, Okazaki T 1992 Centromere protein B assembles human centromeric alpha-satellite DNA at the 17-bp sequence, CENP-B box. J. Cell Biol. 116, 585–596. (10.1083/jcb.116.3.585) 1730770 PMC2289308

[59] Fachinetti D, Han JS, McMahon MA, Ly P, Abdullah A, Wong AJ, Cleveland DW 2015 DNA sequence-specific binding of CENP-B enhances the fidelity of human centromere function. Dev. Cell 33, 314–327. (10.1016/j.devcel.2015.03.020) 25942623 PMC4421092

[60] Tanaka Y, Nureki O, Kurumizaka H, Fukai S, Kawaguchi S, Ikuta M, Iwahara J, Okazaki T, Yokoyama S 2001 Crystal structure of the CENP-B protein-DNA complex: the DNA-binding domains of CENP-B induce kinks in the CENP-B box DNA. EMBO J. 20, 6612–6618. (10.1093/emboj/20.23.6612) 11726497 PMC125324

[61] Kapoor M, Montes de Oca Luna R, Liu G, Lozano G, Cummings C, Mancini M, Ouspenski I, Brinkley BR, May GS 1998 The cenpB gene is not essential in mice. Chromosoma 107, 570–576. (10.1007/s004120050343) 9933410

[62] Hudson DF et al 1998 Centromere protein B null mice are mitotically and meiotically normal but have lower body and testis weights. J. Cell Biol. 141, 309–319. (10.1083/jcb.141.2.309) 9548711 PMC2148459

[63] Perez-Castro AV, Shamanski FL, Meneses JJ, Lovato TL, Vogel KG, Moyzis RK, Pedersen R 1998 Centromeric protein B null mice are viable with no apparent abnormalities. Dev. Biol. 201, 135–143. (10.1006/dbio.1998.9005) 9740654

[64] Ohzeki J, Nakano M, Okada T, Masumoto H 2002 CENP-B box is required for de novo centromere chromatin assembly on human alphoid DNA. J. Cell Biol. 159, 765–775. (10.1083/jcb.200207112) 12460987 PMC2173396

[65] Okada T, Ohzeki J, Nakano M, Yoda K, Brinkley WR, Larionov V, Masumoto H 2007 CENP-B controls centromere formation depending on the chromatin context. Cell 131, 1287–1300. (10.1016/j.cell.2007.10.045) 18160038

[66] Logsdon GA, Gambogi CW, Liskovykh MA, Barrey EJ, Larionov V, Miga KH, Heun P, Black BE 2019 Human artificial chromosomes that bypass centromeric DNA. Cell 178, 624–639.e19. (10.1016/j.cell.2019.06.006) 31348889 PMC6657561

[67] McNulty SM, Sullivan LL, Sullivan BA 2017 Human centromeres produce chromosome-specific and array-specific alpha satellite transcripts that are complexed with CENP-A and CENP-C. Dev. Cell 42, 226–240.e6. (10.1016/j.devcel.2017.07.001) 28787590 PMC5568664

[68] Shukla M, Tong P, White SA, Singh PP, Reid AM, Catania S, Pidoux AL, Allshire RC 2018 Centromere DNA destabilizes H3 nucleosomes to promote CENP-A deposition during the cell cycle. Curr. Biol. 28, 3924–3936.e4. (10.1016/j.cub.2018.10.049) 30503616 PMC6303189

[69] Bobkov GOM, Gilbert N, Heun P 2018 Centromere transcription allows CENP-A to transit from chromatin association to stable incorporation. J. Cell Biol. 217, 1957–1972. (10.1083/jcb.201611087) 29626011 PMC5987708

[70] Srivastava S, Foltz DR 2018 Posttranslational modifications of CENP-A: marks of distinction. Chromosoma 127, 279–290. (10.1007/s00412-018-0665-x) 29569072 PMC6082721

[71] Fachinetti D et al 2013 A two-step mechanism for epigenetic specification of centromere identity and function. Nat. Cell Biol. 15, 1056–1066. (10.1038/ncb2805) 23873148 PMC4418506

[72] Liu S-T, Rattner JB, Jablonski SA, Yen TJ 2006 Mapping the assembly pathways that specify formation of the trilaminar kinetochore plates in human cells. J. Cell Biol. 175, 41–53. (10.1083/jcb.200606020) 17030981 PMC2064494

[73] Palmer DK, Margolis RL 1985 Kinetochore components recognized by human autoantibodies are present on mononucleosomes. Mol. Cell. Biol. 5, 173–186. (10.1128/mcb.5.1.173) 2580229 PMC366692

[74] Sullivan KF, Hechenberger M, Masri K 1994 Human CENP-A contains a histone H3 related histone fold domain that is required for targeting to the centromere. J. Cell Biol. 127, 581–592. (10.1083/jcb.127.3.581) 7962047 PMC2120219

[75] Zinkowski RP, Meyne J, Brinkley BR 1991 The centromere–kinetochore complex: a repeat subunit model. J. Cell Biol. 113, 1091–1110. (10.1083/jcb.113.5.1091) 1828250 PMC2289018

[76] Ribeiro SA, Vagnarelli P, Dong Y, Hori T, McEwen BF, Fukagawa T, Flors C, Earnshaw WC 2010 A super-resolution map of the vertebrate kinetochore. Proc. Natl Acad. Sci. USA 107, 10 484–10 489. (10.1073/pnas.1002325107) PMC2890832 20483991

[77] Goutte-Gattat D, Shuaib M, Ouararhni K, Gautier T, Skoufias DA, Hamiche A, Dimitrov S 2013 Phosphorylation of the CENP-A amino-terminus in mitotic centromeric chromatin is required for kinetochore function. Proc. Natl Acad. Sci. USA 110, 8579–8584. (10.1073/pnas.1302955110) 23657009 PMC3666736

[78] Black BE, Brock MA, Bédard S, Woods VL, Cleveland DW 2007 An epigenetic mark generated by the incorporation of CENP-A into centromeric nucleosomes. Proc. Natl Acad. Sci. USA 104, 5008–5013. (10.1073/pnas.0700390104) 17360341 PMC1829255

[79] Black BE, Jansen LET, Maddox PS, Foltz DR, Desai AB, Shah JV, Cleveland DW 2007 Centromere identity maintained by nucleosomes assembled with histone H3 containing the CENP-A targeting domain. Mol. Cell 25, 309–322. (10.1016/j.molcel.2006.12.018) 17244537

[80] Black BE, Foltz DR, Chakravarthy S, Luger K, Woods VL, Cleveland DW 2004 Structural determinants for generating centromeric chromatin. Nature 430, 578–582. (10.1038/nature02766) 15282608

[81] Carroll CW, Milks KJ, Straight AF 2010 Dual recognition of CENP-A nucleosomes is required for centromere assembly. J. Cell Biol. 189, 1143–1155. (10.1083/jcb.201001013) 20566683 PMC2894454

[82] Carroll CW, Silva MCC, Godek KM, Jansen LET, Straight AF 2009 Centromere assembly requires the direct recognition of CENP-A nucleosomes by CENP-N. Nat. Cell Biol. 11, 896–902. (10.1038/ncb1899) 19543270 PMC2704923

[83] Moree B, Meyer CB, Fuller CJ, Straight AF 2011 CENP-C recruits M18BP1 to centromeres to promote CENP-A chromatin assembly. J. Cell Biol. 194, 855–871. (10.1083/jcb.201106079) 21911481 PMC3207292

[84] Logsdon GA, Barrey EJ, Bassett EA, DeNizio JE, Guo LY, Panchenko T, Dawicki-McKenna JM, Heun P, Black BE 2015 Both tails and the centromere targeting domain of CENP-A are required for centromere establishment. J. Cell Biol. 208, 521–531. (10.1083/jcb.201412011) 25713413 PMC4347640

[85] Kato H, Jiang J, Zhou B-R, Rozendaal M, Feng H, Ghirlando R, Xiao TS, Straight AF, Bai Y 2013 A conserved mechanism for centromeric nucleosome recognition by centromere protein CENP-C. Science 340, 1110–1113. (10.1126/science.1235532) 23723239 PMC3763809

[86] Allu PK, Dawicki-McKenna JM, Van Eeuwen T, Slavin M, Braitbard M, Xu C, Kalisman N, Murakami K, Black BE. 2019 Structure of the human core centromeric nucleosome complex. Curr. Biol. 29, 2625–2639.e5. (10.1016/j.cub.2019.06.062) 31353180 PMC6702948

[87] Ali-Ahmad A, Bilokapić S, Schäfer IB, Halić M, Sekulić N 2019 CENP-C unwraps the human CENP-A nucleosome through the H2A C-terminal tail. EMBO Rep. 20, e48913 (10.15252/embr.201948913) 31475439 PMC6776904

[88] Sekulic N, Bassett EA, Rogers DJ, Black BE 2010 The structure of (CENP-A-H4)_2_ reveals physical features that mark centromeres. Nature 467, 347–351. (10.1038/nature09323) 20739937 PMC2946842

[89] Tachiwana H et al 2011 Crystal structure of the human centromeric nucleosome containing CENP-A. Nature 476, 232–235. (10.1038/nature10258) 21743476

[90] Hasson D, Panchenko T, Salimian KJ, Salman MU, Sekulic N, Alonso A, Warburton PE, Black BE 2013 The octamer is the major form of CENP-A nucleosomes at human centromeres. Nat. Struct. Mol. Biol. 20, 687–695. (10.1038/nsmb.2562) 23644596 PMC3760417

[91] Zhou B-R, Yadav KNS, Borgnia M, Hong J, Cao B, Olins AL, Olins DE, Bai Y, Zhang P 2019 Atomic resolution cryo-EM structure of a native-like CENP-A nucleosome aided by an antibody fragment. Nat. Commun. 10, 2301 (10.1038/s41467-019-10247-4) 31127102 PMC6534667

[92] Dalal Y, Wang H, Lindsay S, Henikoff S 2007 Tetrameric structure of centromeric nucleosomes in interphase *Drosophila* cells. PLoS Biol. 5, e218 (10.1371/journal.pbio.0050218) 17676993 PMC1933458

[93] Williams JS, Hayashi T, Yanagida M, Russell P 2009 Fission yeast Scm3 mediates stable assembly of Cnp1/CENP-A into centromeric chromatin. Mol. Cell 33, 287–298. (10.1016/j.molcel.2009.01.017) 19217403 PMC2677390

[94] Furuyama T, Henikoff S 2009 Centromeric nucleosomes induce positive DNA supercoils. Cell 138, 104–113. (10.1016/j.cell.2009.04.049) 19596238 PMC2725230

[95] Mizuguchi G, Xiao H, Wisniewski J, Smith MM, Wu C 2007 Nonhistone Scm3 and histones CenH3-H4 assemble the core of centromere-specific nucleosomes. Cell 129, 1153–1164. (10.1016/j.cell.2007.04.026) 17574026

[96] Panchenko T et al 2011 Replacement of histone H3 with CENP-A directs global nucleosome array condensation and loosening of nucleosome superhelical termini. Proc. Natl Acad. Sci. USA 108, 16 588–16 593. (10.1073/pnas.1113621108) PMC3189058 21949362

[97] Falk SJ et al 2015 CENP-C reshapes and stabilizes CENP-A nucleosomes at the centromere. Science 348, 699–703. (10.1126/science.1259308) 25954010 PMC4610723

[98] Conde e Silva N, Black BE, Sivolob A, Filipski J, Cleveland DW, Prunell A 2007 CENP-A-containing nucleosomes: easier disassembly versus exclusive centromeric localization. J. Mol. Biol. 370, 555–573. (10.1016/j.jmb.2007.04.064) 17524417

[99] Bilokapic S, Strauss M, Halic M 2018 Structural rearrangements of the histone octamer translocate DNA. Nat. Commun. 9, 1330 (10.1038/s41467-018-03677-z) 29626188 PMC5889399

[100] Falk SJ, Lee J, Sekulic N, Sennett MA, Lee T-H, Black BE 2016 CENP-C directs a structural transition of CENP-A nucleosomes mainly through sliding of DNA gyres. Nat. Struct. Mol. Biol. 23, 204–208. (10.1038/nsmb.3175) 26878239 PMC4893769

[101] Jansen LET, Black BE, Foltz DR, Cleveland DW 2007 Propagation of centromeric chromatin requires exit from mitosis. J. Cell Biol. 176, 795–805. (10.1083/jcb.200701066) 17339380 PMC2064054

[102] Bodor DL, Valente LP, Mata JF, Black BE, Jansen LET 2013 Assembly in G1 phase and long-term stability are unique intrinsic features of CENP-A nucleosomes. Mol. Biol. Cell 24, 923–932. (10.1091/mbc.E13-01-0034) 23363600 PMC3608502

[103] Nechemia-Arbely Y et al 2017 Human centromeric CENP-A chromatin is a homotypic, octameric nucleosome at all cell cycle points. J. Cell Biol. 216, 607–621. (10.1083/jcb.201608083) 28235947 PMC5350519

[104] Schuh M, Lehner CF, Heidmann S 2007 Incorporation of *Drosophila* CID/CENP-A and CENP-C into centromeres during early embryonic anaphase. Curr. Biol. 17, 237–243. (10.1016/j.cub.2006.11.051) 17222555

[105] Dunleavy EM, Almouzni G, Karpen GH 2011 H3.3 is deposited at centromeres in S phase as a placeholder for newly assembled CENP-A in G₁ phase. Nucleus 2, 146–157. (10.4161/nucl.2.2.15211) 21738837 PMC3127096

[106] Foltz DR, Jansen LET, Bailey AO, Yates JR, Bassett EA, Wood S, Black BE, Cleveland DW 2009 Centromere-specific assembly of CENP-A nucleosomes is mediated by HJURP. Cell 137, 472–484. (10.1016/j.cell.2009.02.039) 19410544 PMC2747366

[107] Dunleavy EM, Roche D, Tagami H, Lacoste N, Ray-Gallet D, Nakamura Y, Daigo Y, Nakatani Y, Almouzni-Pettinotti G 2009 HJURP is a cell-cycle-dependent maintenance and deposition factor of CENP-A at centromeres. Cell 137, 485–497. (10.1016/j.cell.2009.02.040) 19410545

[108] Zasadzińska E et al 2018 Inheritance of CENP-A nucleosomes during DNA replication requires HJURP. Dev. Cell 47, 348–362.e7. (10.1016/j.devcel.2018.09.003) 30293838 PMC6219920

[109] Shuaib M, Ouararhni K, Dimitrov S, Hamiche A 2010 HJURP binds CENP-A via a highly conserved N-terminal domain and mediates its deposition at centromeres. Proc. Natl Acad. Sci. USA 107, 1349–1354. (10.1073/pnas.0913709107) 20080577 PMC2824361

[110] Barnhart MC, Kuich PHJL, Stellfox ME, Ward JA, Bassett EA, Black BE, Foltz DR 2011 HJURP is a CENP-A chromatin assembly factor sufficient to form a functional de novo kinetochore. J. Cell Biol. 194, 229–243. (10.1083/jcb.201012017) 21768289 PMC3144403

[111] Hori T, Shang W-H, Takeuchi K, Fukagawa T 2013 The CCAN recruits CENP-A to the centromere and forms the structural core for kinetochore assembly. J. Cell Biol. 200, 45–60. (10.1083/jcb.201210106) 23277427 PMC3542802

[112] Hu H et al 2011 Structure of a CENP-A-histone H4 heterodimer in complex with chaperone HJURP. Genes Dev. 25, 901–906. (10.1101/gad.2045111) 21478274 PMC3084024

[113] Stankovic A et al 2017 A dual inhibitory mechanism sufficient to maintain cell-cycle-restricted CENP-A assembly. Mol. Cell 65, 231–246. (10.1016/j.molcel.2016.11.021) 28017591 PMC5250512

[114] Fujita Y, Hayashi T, Kiyomitsu T, Toyoda Y, Kokubu A, Obuse C, Yanagida M 2007 Priming of centromere for CENP-A recruitment by human hMis18α, hMis18β, and M18BP1. Dev. Cell 12, 17–30. (10.1016/j.devcel.2006.11.002) 17199038

[115] Maddox PS, Hyndman F, Monen J, Oegema K, Desai A 2007 Functional genomics identifies a Myb domain-containing protein family required for assembly of CENP-A chromatin. J. Cell Biol. 176, 757–763. (10.1083/jcb.200701065) 17339379 PMC2064049

[116] Spiller F, Medina-Pritchard B, Abad MA, Wear MA, Molina O, Earnshaw WC, Jeyaprakash AA 2017 Molecular basis for Cdk1-regulated timing of Mis18 complex assembly and CENP-A deposition. EMBO Rep. 18, 894–905. (10.15252/embr.201643564) 28377371 PMC5452045

[117] Pan D, Klare K, Petrovic A, Take A, Walstein K, Singh P, Rondelet A, Bird AW, Musacchio A 2017 CDK-regulated dimerization of M18BP1 on a Mis18 hexamer is necessary for CENP-A loading. Elife 6, e23352 (10.7554/eLife.23352) 28059702 PMC5245964

[118] Korntner-Vetter M, Lefèvre S, Hu X-W, George R, Singleton MR 2019 Subunit interactions and arrangements in the fission yeast Mis16-Mis18-Mis19 complex. Life Sci. Alliance 2, e201900408 (10.26508/lsa.201900408) 31371524 PMC6677171

[119] Hayashi T, Fujita Y, Iwasaki O, Adachi Y, Takahashi K, Yanagida M 2004 Mis16 and Mis18 are required for CENP-A loading and histone deacetylation at centromeres. Cell 118, 715–729. (10.1016/j.cell.2004.09.002) 15369671

[120] Hayashi T, Ebe M, Nagao K, Kokubu A, Sajiki K, Yanagida M 2014 *Schizosaccharomyces pombe* centromere protein Mis19 links Mis16 and Mis18 to recruit CENP-A through interacting with NMD factors and the SWI/SNF complex. Genes Cells 19, 541–554. (10.1111/gtc.12152) 24774534

[121] Hirai H, Arai K, Kariyazono R, Yamamoto M, Sato M 2014 The kinetochore protein Kis1/Eic1/Mis19 ensures the integrity of mitotic spindles through maintenance of kinetochore factors Mis6/CENP-I and CENP-A. PLoS ONE 9, e111905 (10.1371/journal.pone.0111905) 25375240 PMC4222959

[122] Subramanian L, Toda NRT, Rappsilber J, Allshire RC 2014 Eic1 links Mis18 with the CCAN/Mis6/Ctf19 complex to promote CENP-A assembly. Open Biol. 4, 140043 (10.1098/rsob.140043) 24789708 PMC4043117

[123] Dambacher S et al 2012 CENP-C facilitates the recruitment of M18BP1 to centromeric chromatin. Nucleus 3, 101–110. (10.4161/nucl.18955) 22540025 PMC3337164

[124] Subramanian L, Medina-Pritchard B, Barton R, Spiller F, Kulasegaran-Shylini R, Radaviciute G, Allshire RC, Arockia Jeyaprakash A 2016 Centromere localization and function of Mis18 requires Yippee-like domain-mediated oligomerization. EMBO Rep. 17, 496–507. (10.15252/embr.201541520) 26921242 PMC4818781

[125] Medina-Pritchard B, Lazou V, Zou J, Byron O, Abad MA, Rappsilber J, Heun P, Jeyaprakash AA 2020 Structural basis for centromere maintenance by *Drosophila* CENP-A chaperone CAL1. EMBO J. 39, e103234 (10.15252/embj.2019103234) 32134144 PMC7110144

[126] Schittenhelm RB, Althoff F, Heidmann S, Lehner CF 2010 Detrimental incorporation of excess Cenp-A/Cid and Cenp-C into *Drosophila* centromeres is prevented by limiting amounts of the bridging factor Cal1. J. Cell Sci. 123, 3768–3779. (10.1242/jcs.067934) 20940262

[127] Phansalkar R, Lapierre P, Mellone BG 2012 Evolutionary insights into the role of the essential centromere protein CAL1 in *Drosophila*. Chromosome Res. 20, 493–504. (10.1007/s10577-012-9299-7) 22820845

[128] Chen C-C, Dechassa ML, Bettini E, Ledoux MB, Belisario C, Heun P, Luger K, Mellone BG 2014 CAL1 is the *Drosophila* CENP-A assembly factor. J. Cell Biol. 204, 313–329. (10.1083/jcb.201305036) 24469636 PMC3912524

[129] Tanaka K, Chang HL, Kagami A, Watanabe Y 2009 CENP-C functions as a scaffold for effectors with essential kinetochore functions in mitosis and meiosis. Dev. Cell 17, 334–343. (10.1016/j.devcel.2009.08.004) 19758558

[130] Klare K, Weir JR, Basilico F, Zimniak T, Massimiliano L, Ludwigs N, Herzog F, Musacchio A 2015 CENP-C is a blueprint for constitutive centromere-associated network assembly within human kinetochores. J. Cell Biol. 210, 11–22. (10.1083/jcb.201412028) 26124289 PMC4494010

[131] Watanabe R, Hara M, Okumura E-I, Hervé S, Fachinetti D, Ariyoshi M, Fukagawa T 2019 CDK1-mediated CENP-C phosphorylation modulates CENP-A binding and mitotic kinetochore localization. J. Cell Biol. 218, 4042–4062. (10.1083/jcb.201907006) 31676716 PMC6891089

[132] Guo LY et al 2017 Centromeres are maintained by fastening CENP-A to DNA and directing an arginine anchor-dependent nucleosome transition. Nat. Commun. 8, 15775 (10.1038/ncomms15775) 28598437 PMC5472775

[133] Xiao H et al 2017 Molecular basis of CENP-C association with the CENP-A nucleosome at yeast centromeres. Genes Dev. 31, 1958–1972. (10.1101/gad.304782.117) 29074736 PMC5710141

[134] Chik JK, Moiseeva V, Goel PK, Meinen BA, Koldewey P, An S, Mellone BG, Subramanian L, Cho U-S 2019 Structures of CENP-C cupin domains at regional centromeres reveal unique patterns of dimerization and recruitment functions for the inner pocket. J. Biol. Chem. 294, 14 119–14 134. (10.1074/jbc.RA119.008464) PMC6755791 31366733

[135] Pentakota S et al 2017 Decoding the centromeric nucleosome through CENP-N. Elife 6, e33442 (10.7554/eLife.33442) 29280735 PMC5777823

[136] Chittori S, Hong J, Saunders H, Feng H, Ghirlando R, Kelly AE, Bai Y, Subramaniam S 2018 Structural mechanisms of centromeric nucleosome recognition by the kinetochore protein CENP-N. Science 359, 339–343. (10.1126/science.aar2781) 29269420 PMC6292214

[137] Tian T et al 2018 Molecular basis for CENP-N recognition of CENP-A nucleosome on the human kinetochore. Cell Res. 28, 374–378. (10.1038/cr.2018.13) 29350209 PMC5835772

[138] Luger K, Mäder AW, Richmond RK, Sargent DF, Richmond TJ. 1997 Crystal structure of the nucleosome core particle at 2.8 Å resolution. Nature 389, 251–260. (10.1038/38444) 9305837

[139] Weir JR et al 2016 Insights from biochemical reconstitution into the architecture of human kinetochores. Nature 537, 249–253. (10.1038/nature19333) 27580032

[140] Meluh PB, Koshland D 1995 Evidence that the MIF2 gene of *Saccharomyces cerevisiae* encodes a centromere protein with homology to the mammalian centromere protein CENP-C. Mol. Biol. Cell 6, 793–807. (10.1091/mbc.6.7.793) 7579695 PMC301241

[141] Ortiz J, Stemmann O, Rank S, Lechner J 1999 A putative protein complex consisting of Ctf19, Mcm21, and Okp1 represents a missing link in the budding yeast kinetochore. Genes Dev. 13, 1140–1155. (10.1101/gad.13.9.1140) PMC316948 10323865

[142] Westermann S, Schleiffer A 2013 Family matters: structural and functional conservation of centromere-associated proteins from yeast to humans. Trends Cell Biol. 23, 260–269. (10.1016/j.tcb.2013.01.010) 23481674

[143] Schleiffer A, Maier M, Litos G, Lampert F, Hornung P, Mechtler K, Westermann S 2012 CENP-T proteins are conserved centromere receptors of the Ndc80 complex. Nat. Cell Biol. 14, 604–613. (10.1038/ncb2493) 22561346

[144] Yan K, Yang J, Zhang Z, McLaughlin SH, Chang L, Fasci D, Ehrenhofer-Murray AE, Heck AJR, Barford D 2019 Structure of the inner kinetochore CCAN complex assembled onto a centromeric nucleosome. Nature 574, 278–282. (10.1038/s41586-019-1609-1) 31578520 PMC6859074

[145] Hinshaw SM, Harrison SC 2019 The structure of the Ctf19c/CCAN from budding yeast. Elife 8, e44239 (10.7554/eLife.44239) 30762520 PMC6407923

[146] Hinshaw SM, Dates AN, Harrison SC 2019 The structure of the yeast Ctf3 complex. Elife 8, e48215 (10.7554/eLife.48215) 31194673 PMC6602579

[147] Basilico F et al 2014 The pseudo GTPase CENP-M drives human kinetochore assembly. Elife 3, e02978 (10.7554/eLife.02978) 25006165 PMC4080450

[148] Nishino T, Takeuchi K, Gascoigne KE, Suzuki A, Hori T, Oyama T, Morikawa K, Cheeseman IM, Fukagawa T 2012 CENP-T-W-S-X forms a unique centromeric chromatin structure with a histone-like fold. Cell 148, 487–501. (10.1016/j.cell.2011.11.061) 22304917 PMC3277711

[149] Lowary PT, Widom J 1998 New DNA sequence rules for high affinity binding to histone octamer and sequence-directed nucleosome positioning. J. Mol. Biol. 276, 19–42. (10.1006/jmbi.1997.1494) 9514715

[150] Hornung P et al 2014 A cooperative mechanism drives budding yeast kinetochore assembly downstream of CENP-A. J. Cell Biol. 206, 509–524. (10.1083/jcb.201403081) 25135934 PMC4137059

[151] Tachiwana H, Müller S, Blümer J, Klare K, Musacchio A, Almouzni G 2015 HJURP involvement in *de novo* CenH3(CENP-A) and CENP-C recruitment. Cell Rep. 11, 22–32. (10.1016/j.celrep.2015.03.013) 25843710

[152] Wan X et al 2009 Protein architecture of the human kinetochore microtubule attachment site. Cell 137, 672–684. (10.1016/j.cell.2009.03.035) 19450515 PMC2699050

[153] Hu L et al 2019 Structural analysis of fungal CENP-H/I/K homologs reveals a conserved assembly mechanism underlying proper chromosome alignment. Nucleic Acids Res. 47, 468–479. (10.1093/nar/gky1108) 30407575 PMC6326798

[154] Yan Z et al 2010 A histone-fold complex and FANCM form a conserved DNA-remodeling complex to maintain genome stability. Mol. Cell 37, 865–878. (10.1016/j.molcel.2010.01.039) 20347428 PMC2847587

[155] Singh TR et al 2010 MHF1-MHF2, a histone-fold-containing protein complex, participates in the Fanconi anemia pathway via FANCM. Mol. Cell 37, 879–886. (10.1016/j.molcel.2010.01.036) 20347429 PMC2848122

[156] Huang M, Kim JM, Shiotani B, Yang K, Zou L, D'Andrea AD 2010 The FANCM/FAAP24 complex is required for the DNA interstrand crosslink-induced checkpoint response. Mol. Cell 39, 259–268. (10.1016/j.molcel.2010.07.005) 20670894 PMC2928996

[157] Prendergast L, van Vuuren C, Kaczmarczyk A, Doering V, Hellwig D, Quinn N, Hoischen C, Diekmann S, Sullivan KF. 2011 Premitotic assembly of human CENPs -T and -W switches centromeric chromatin to a mitotic state. PLoS Biol. 9, e1001082 (10.1371/journal.pbio.1001082) 21695110 PMC3114758

[158] Samejima I, Spanos C, Alves F de L, Hori T, Perpelescu M, Zou J, Rappsilber J, Fukagawa T, Earnshaw WC 2015 Whole-proteome genetic analysis of dependencies in assembly of a vertebrate kinetochore. J. Cell Biol. 211, 1141–1156. (10.1083/jcb.201508072) 26668330 PMC4687880

[159] Folco HD, Campbell CS, May KM, Espinoza CA, Oegema K, Hardwick KG, Grewal SIS, Desai A 2015 The CENP-A N-tail confers epigenetic stability to centromeres via the CENP-T branch of the CCAN in fission yeast. Curr. Biol. 25, 348–356. (10.1016/j.cub.2014.11.060) 25619765 PMC4318777

[160] Takeuchi K, Nishino T, Mayanagi K, Horikoshi N, Osakabe A, Tachiwana H, Hori T, Kurumizaka H, Fukagawa T 2014 The centromeric nucleosome-like CENP-T-W-S-X complex induces positive supercoils into DNA. Nucleic Acids Res. 42, 1644–1655. (10.1093/nar/gkt1124) 24234442 PMC3919578

[161] Pekgöz Altunkaya G, Malvezzi F, Demianova Z, Zimniak T, Litos G, Weissmann F, Mechtler K, Herzog F, Westermann S 2016 CCAN assembly configures composite binding interfaces to promote cross-linking of Ndc80 complexes at the kinetochore. Curr. Biol. 26, 2370–2378. (10.1016/j.cub.2016.07.005) 27524485

[162] Schmitzberger F, Harrison SC 2012 RWD domain: a recurring module in kinetochore architecture shown by a Ctf19-Mcm21 complex structure. EMBO Rep. 13, 216–222. (10.1038/embor.2012.1) 22322944 PMC3323139

[163] Schmitzberger F, Richter MM, Gordiyenko Y, Robinson CV, Dadlez M, Westermann S 2017 Molecular basis for inner kinetochore configuration through RWD domain-peptide interactions. EMBO J. 36, 3458–3482. (10.15252/embj.201796636) 29046335 PMC5709738

[164] Bancroft J, Auckland P, Samora CP, McAinsh AD 2015 Chromosome congression is promoted by CENP-Q- and CENP-E-dependent pathways. J. Cell Sci. 128, 171–184. (10.1242/jcs.163659) 25395579 PMC4282051

[165] Kang YH et al 2006 Self-regulated Plk1 recruitment to kinetochores by the Plk1-PBIP1 interaction is critical for proper chromosome segregation. Mol. Cell 24, 409–422. (10.1016/j.molcel.2006.10.016) 17081991

[166] Hori T, Okada M, Maenaka K, Fukagawa T 2008 CENP-O class proteins form a stable complex and are required for proper kinetochore function. Mol. Biol. Cell 19, 843–854. (10.1091/mbc.E07-06-0556) 18094054 PMC2262965

[167] Hua S, Wang Z, Jiang K, Huang Y, Ward T, Zhao L, Dou Z, Yao X 2011 CENP-U cooperates with Hec1 to orchestrate kinetochore–microtubule attachment. J. Biol. Chem. 286, 1627–1638. (10.1074/jbc.M110.174946) 21056971 PMC3020771

[168] Amaro AC, Samora CP, Holtackers R, Wang E, Kingston IJ, Alonso M, Lampson M, McAinsh AD, Meraldi P 2010 Molecular control of kinetochore–microtubule dynamics and chromosome oscillations. Nat. Cell Biol. 12, 319–329. (10.1038/ncb2033) 20228811 PMC2909587

[169] He X, Rines DR, Espelin CW, Sorger PK 2001 Molecular analysis of kinetochore–microtubule attachment in budding yeast. Cell 106, 195–206. (10.1016/s0092-8674(01)00438-x) 11511347

[170] Janke C, Ortiz J, Lechner J, Shevchenko A, Shevchenko A, Magiera MM, Schramm C, Schiebel E 2001 The budding yeast proteins Spc24p and Spc25p interact with Ndc80p and Nuf2p at the kinetochore and are important for kinetochore clustering and checkpoint control. EMBO J. 20, 777–791. (10.1093/emboj/20.4.777) 11179222 PMC145434

[171] McCleland ML, Gardner RD, Kallio MJ, Daum JR, Gorbsky GJ, Burke DJ, Stukenberg PT 2003 The highly conserved Ndc80 complex is required for kinetochore assembly, chromosome congression, and spindle checkpoint activity. Genes Dev. 17, 101–114. (10.1101/gad.1040903) 12514103 PMC195965

[172] Cheeseman IM, Hori T, Fukagawa T, Desai A 2008 KNL1 and the CENP-H/I/K complex coordinately direct kinetochore assembly in vertebrates. Mol. Biol. Cell 19, 587–594. (10.1091/mbc.e07-10-1051) 18045986 PMC2230600

[173] Malvezzi F, Litos G, Schleiffer A, Heuck A, Mechtler K, Clausen T, Westermann S 2013 A structural basis for kinetochore recruitment of the Ndc80 complex via two distinct centromere receptors. EMBO J. 32, 409–423. (10.1038/emboj.2012.356) 23334295 PMC3567489

[174] Huis in ‘t Veld PJ, Jeganathan S, Petrovic A, Singh P, John J, Krenn V, Weissmann F, Bange T, Musacchio A 2016 Molecular basis of outer kinetochore assembly on CENP-T. Elife 5, e21007 (10.7554/eLife.21007) 28012276 PMC5241120

[175] Gascoigne KE, Cheeseman IM 2013 CDK-dependent phosphorylation and nuclear exclusion coordinately control kinetochore assembly state. J. Cell Biol. 201, 23–32. (10.1083/jcb.201301006) 23530067 PMC3613690

[176] Bharadwaj R, Qi W, Yu H 2004 Identification of two novel components of the human NDC80 kinetochore complex. J. Biol. Chem. 279, 13 076–13 085. (10.1074/jbc.M310224200) 14699129

[177] DeLuca JG, Gall WE, Ciferri C, Cimini D, Musacchio A, Salmon ED 2006 Kinetochore microtubule dynamics and attachment stability are regulated by Hec1. Cell 127, 969–982. (10.1016/j.cell.2006.09.047) 17129782

[178] Wei RR, Schnell JR, Larsen NA, Sorger PK, Chou JJ, Harrison SC 2006 Structure of a central component of the yeast kinetochore: the Spc24p/Spc25p globular domain. Structure 14, 1003–1009. (10.1016/j.str.2006.04.007) 16765893

[179] Ciferri C et al*.* 2005 Architecture of the human Ndc80-Hec1 complex, a critical constituent of the outer kinetochore. J. Biol. Chem. 280, 29 088–29 095. (10.1074/jbc.M504070200) 15961401

[180] Ciferri C et al 2008 Implications for kinetochore–microtubule attachment from the structure of an engineered Ndc80 complex. Cell 133, 427–439. (10.1016/j.cell.2008.03.020) 18455984 PMC4754795

[181] Nishino T, Rago F, Hori T, Tomii K, Cheeseman IM, Fukagawa T 2013 CENP-T provides a structural platform for outer kinetochore assembly. EMBO J. 32, 424–436. (10.1038/emboj.2012.348) 23334297 PMC3567495

[182] Alushin GM, Ramey VH, Pasqualato S, Ball DA, Grigorieff N, Musacchio A, Nogales E 2010 The Ndc80 kinetochore complex forms oligomeric arrays along microtubules. Nature 467, 805–810. (10.1038/nature09423) 20944740 PMC2957311

[183] Valverde R, Ingram J, Harrison SC 2016 Conserved tetramer junction in the kinetochore Ndc80 complex. Cell Rep. 17, 1915–1922. (10.1016/j.celrep.2016.10.065) 27851957 PMC5131873

[184] Alushin GM, Musinipally V, Matson D, Tooley J, Stukenberg PT, Nogales E 2012 Multimodal microtubule binding by the Ndc80 kinetochore complex. Nat. Struct. Mol. Biol. 19, 1161–1167. (10.1038/nsmb.2411) 23085714 PMC3492541

[185] Guimaraes GJ, Dong Y, McEwen BF, DeLuca JG 2008 Kinetochore–microtubule attachment relies on the disordered N-terminal tail domain of Hec1. Curr. Biol. 18, 1778–1784. (10.1016/j.cub.2008.08.012) 19026543 PMC2753282

[186] Miller SA, Johnson ML, Stukenberg PT 2008 Kinetochore attachments require an interaction between unstructured tails on microtubules and Ndc80(Hec1). Curr. Biol. 18, 1785–1791. (10.1016/j.cub.2008.11.007) 19026542 PMC3145211

[187] Sundin LJR, Guimaraes GJ, Deluca JG 2011 The NDC80 complex proteins Nuf2 and Hec1 make distinct contributions to kinetochore–microtubule attachment in mitosis. Mol. Biol. Cell 22, 759–768. (10.1091/mbc.E10-08-0671) 21270439 PMC3057701

[188] Umbreit NT, Gestaut DR, Tien JF, Vollmar BS, Gonen T, Asbury CL, Davis TN 2012 The Ndc80 kinetochore complex directly modulates microtubule dynamics. Proc. Natl Acad. Sci. USA 109, 16 113–16 118. (10.1073/pnas.1209615109) PMC3479545 22908300

[189] Volkov VA, Huis in ‘t Veld PJ, Dogterom M, Musacchio A 2018 Multivalency of NDC80 in the outer kinetochore is essential to track shortening microtubules and generate forces. Elife 7, e36764 (10.7554/eLife.36764) 29629870 PMC5940359

[190] Huis in ‘t Veld PJ, Volkov VA, Stender ID, Musacchio A, Dogterom M 2019 Molecular determinants of the Ska-Ndc80 interaction and their influence on microtubule tracking and force-coupling. Elife 8, e49539 (10.7554/eLife.49539) 31804178 PMC6927755

[191] Scarborough EA, Davis TN, Asbury CL 2019 Tight bending of the Ndc80 complex provides intrinsic regulation of its binding to microtubules. Elife 8, 44489 (10.7554/eLife.44489) PMC6516834 31045495

[192] Petrovic A et al 2010 The MIS12 complex is a protein interaction hub for outer kinetochore assembly. J. Cell Biol. 190, 835–852. (10.1083/jcb.201002070) 20819937 PMC2935574

[193] Petrovic A et al 2016 Structure of the MIS12 complex and molecular basis of its interaction with CENP-C at human kinetochores. Cell 167, 1028–1040.e15. (10.1016/j.cell.2016.10.005) 27881301 PMC5101189

[194] Dimitrova YN, Jenni S, Valverde R, Khin Y, Harrison SC 2016 Structure of the MIND complex defines a regulatory focus for yeast kinetochore assembly. Cell 167, 1014–1027.e12. (10.1016/j.cell.2016.10.011) 27881300 PMC5856483

[195] Maskell DP, Hu X-W, Singleton MR 2010 Molecular architecture and assembly of the yeast kinetochore MIND complex. J. Cell Biol. 190, 823–834. (10.1083/jcb.201002059) 20819936 PMC2935576

[196] Screpanti E, De Antoni A, Alushin GM, Petrovic A, Melis T, Nogales E, Musacchio A. 2011 Direct binding of Cenp-C to the Mis12 complex joins the inner and outer kinetochore. Curr. Biol. 21, 391–398. (10.1016/j.cub.2010.12.039) 21353556 PMC3074538

[197] Gascoigne KE, Takeuchi K, Suzuki A, Hori T, Fukagawa T, Cheeseman IM 2011 Induced ectopic kinetochore assembly bypasses the requirement for CENP-A nucleosomes. Cell 145, 410–422. (10.1016/j.cell.2011.03.031) 21529714 PMC3085131

[198] Petrovic A et al 2014 Modular assembly of RWD domains on the Mis12 complex underlies outer kinetochore organization. Mol. Cell 53, 591–605. (10.1016/j.molcel.2014.01.019) 24530301

[199] Kiyomitsu T, Murakami H, Yanagida M 2011 Protein interaction domain mapping of human kinetochore protein blinkin reveals a consensus motif for binding of spindle assembly checkpoint proteins Bub1 and BubR1. Mol. Cell. Biol. 31, 998–1011. (10.1128/MCB.00815-10) 21199919 PMC3067818

[200] Varma D, Wan X, Cheerambathur D, Gassmann R, Suzuki A, Lawrimore J, Desai A, Salmon ED 2013 Spindle assembly checkpoint proteins are positioned close to core microtubule attachment sites at kinetochores. J. Cell Biol. 202, 735–746. (10.1083/jcb.201304197) 23979716 PMC3760617

[201] Seo D Woo, You S Yeop, Chung W-J, Cho D-H, Kim J-S, Su Oh J 2015 Zwint-1 is required for spindle assembly checkpoint function and kinetochore–microtubule attachment during oocyte meiosis. Sci. Rep. 5, 15431 (10.1038/srep15431) 26486467 PMC4614028

[202] Kasuboski JM et al 2011 Zwint-1 is a novel Aurora B substrate required for the assembly of a dynein-binding platform on kinetochores. Mol. Biol. Cell 22, 3318–3330. (10.1091/mbc.E11-03-0213) 21775627 PMC3172258

[203] Wang H et al 2004 Human Zwint-1 specifies localization of Zeste White 10 to kinetochores and is essential for mitotic checkpoint signaling. J. Biol. Chem. 279, 54 590–54 598. (10.1074/jbc.M407588200) 15485811

[204] Gaitanos TN, Santamaria A, Jeyaprakash AA, Wang B, Conti E, Nigg EA 2009 Stable kinetochore–microtubule interactions depend on the Ska complex and its new component Ska3/C13Orf3. EMBO J. 28, 1442–1452. (10.1038/emboj.2009.96) 19360002 PMC2669960

[205] Schmidt JC et al 2012 The kinetochore-bound Ska1 complex tracks depolymerizing microtubules and binds to curved protofilaments. Dev. Cell 23, 968–980. (10.1016/j.devcel.2012.09.012) 23085020 PMC3500403

[206] Jeyaprakash AA, Santamaria A, Jayachandran U, Chan YW, Benda C, Nigg EA, Conti E 2012 Structural and functional organization of the Ska complex, a key component of the kinetochore–microtubule interface. Mol. Cell 46, 274–286. (10.1016/j.molcel.2012.03.005) 22483620

[207] Abad MA, Zou J, Medina-Pritchard B, Nigg EA, Rappsilber J, Santamaria A, Jeyaprakash AA 2016 Ska3 ensures timely mitotic progression by interacting directly with microtubules and Ska1 microtubule binding domain. Sci. Rep. 6, 34042 (10.1038/srep34042) 27667719 PMC5036024

[208] Abad MA et al 2014 Structural basis for microtubule recognition by the human kinetochore Ska complex. Nat. Commun. 5, 2964 (10.1038/ncomms3964) 24413531 PMC3923297

[209] Monda JK, Whitney IP, Tarasovetc EV, Wilson-Kubalek E, Milligan RA, Grishchuk EL, Cheeseman IM 2017 Microtubule tip tracking by the spindle and kinetochore protein Ska1 requires diverse tubulin-interacting surfaces. Curr. Biol. 27, 3666–3675.e6. (10.1016/j.cub.2017.10.018) 29153323 PMC5726585

[210] Cheeseman IM et al 2001 Implication of a novel multiprotein Dam1p complex in outer kinetochore function. J. Cell Biol. 155, 1137–1145. (10.1083/jcb.200109063) 11756468 PMC2199314

[211] Westermann S, Avila-Sakar A, Wang H-W, Niederstrasser H, Wong J, Drubin DG, Nogales E, Barnes G 2005 Formation of a dynamic kinetochore–microtubule interface through assembly of the Dam1 ring complex. Mol. Cell 17, 277–290. (10.1016/j.molcel.2004.12.019) 15664196

[212] Westermann S, Wang H-W, Avila-Sakar A, Drubin DG, Nogales E, Barnes G 2006 The Dam1 kinetochore ring complex moves processively on depolymerizing microtubule ends. Nature 440, 565–569. (10.1038/nature04409) 16415853

[213] Miranda JJL, De Wulf P, Sorger PK, Harrison SC. 2005 The yeast DASH complex forms closed rings on microtubules. Nat. Struct. Mol. Biol. 12, 138–143. (10.1038/nsmb896) 15640796

[214] Karess R 2005 Rod-Zw10-Zwilch: a key player in the spindle checkpoint. Trends Cell Biol. 15, 386–392. (10.1016/j.tcb.2005.05.003) 15922598

[215] McEwen BF, Chan GK, Zubrowski B, Savoian MS, Sauer MT, Yen TJ 2001 CENP-E is essential for reliable bioriented spindle attachment, but chromosome alignment can be achieved via redundant mechanisms in mammalian cells. Mol. Biol. Cell 12, 2776–2789. (10.1091/mbc.12.9.2776) 11553716 PMC59712

[216] Feng J, Huang H, Yen TJ 2006 CENP-F is a novel microtubule-binding protein that is essential for kinetochore attachments and affects the duration of the mitotic checkpoint delay. Chromosoma 115, 320–329. (10.1007/s00412-006-0049-5) 16601978

[217] Wynne DJ, Funabiki H 2015 Kinetochore function is controlled by a phospho-dependent coexpansion of inner and outer components. J. Cell Biol. 210, 899–916. (10.1083/jcb.201506020) 26347137 PMC4576862

[218] Magidson V, O'Connell CB, Lončarek J, Paul R, Mogilner A, Khodjakov A 2011 The spatial arrangement of chromosomes during prometaphase facilitates spindle assembly. Cell 146, 555–567. (10.1016/j.cell.2011.07.012) 21854981 PMC3291198

[219] Thrower DA, Jordan MA, Wilson L 1996 Modulation of CENP-E organization at kinetochores by spindle microtubule attachment. Cell Motil. Cytoskeleton 35, 121–133. (10.1002/(SICI)1097-0169(1996)35:2<121::AID-CM5>3.0.CO;2-D) 8894282

[220] Magidson V, He J, Ault JG, O'Connell CB, Yang N, Tikhonenko I, McEwen BF, Sui H, Khodjakov A 2016 Unattached kinetochores rather than intrakinetochore tension arrest mitosis in taxol-treated cells. J. Cell Biol. 212, 307–319. (10.1083/jcb.201412139) 26833787 PMC4748573

[221] Wojcik E, Basto R, Serr M, Scaërou F, Karess R, Hays T 2001 Kinetochore dynein: its dynamics and role in the transport of the Rough deal checkpoint protein. Nat. Cell Biol. 3, 1001–1007. (10.1038/ncb1101-1001) 11715021

[222] Basto R, Scaerou F, Mische S, Wojcik E, Lefebvre C, Gomes R, Hays T, Karess R 2004 *In vivo* dynamics of the rough deal checkpoint protein during *Drosophila* mitosis. Curr. Biol. 14, 56–61. (10.1016/j.cub.2003.12.025) 14711415

[223] Howell BJ, McEwen BF, Canman JC, Hoffman DB, Farrar EM, Rieder CL, Salmon ED 2001 Cytoplasmic dynein/dynactin drives kinetochore protein transport to the spindle poles and has a role in mitotic spindle checkpoint inactivation. J. Cell Biol. 155, 1159–1172. (10.1083/jcb.200105093) 11756470 PMC2199338

[224] Mische S, He Y, Ma L, Li M, Serr M, Hays TS 2008 Dynein light intermediate chain: an essential subunit that contributes to spindle checkpoint inactivation. Mol. Biol. Cell 19, 4918–4929. (10.1091/mbc.e08-05-0483) 18799620 PMC2575169

[225] Sivaram MVS, Wadzinski TL, Redick SD, Manna T, Doxsey SJ 2009 Dynein light intermediate chain 1 is required for progress through the spindle assembly checkpoint. EMBO J. 28, 902–914. (10.1038/emboj.2009.38) 19229290 PMC2670866

[226] Varma D, Monzo P, Stehman SA, Vallee RB 2008 Direct role of dynein motor in stable kinetochore-microtubule attachment, orientation, and alignment. J. Cell Biol. 182, 1045–1054. (10.1083/jcb.200710106) 18809721 PMC2542467

[227] Gassmann R, Holland AJ, Varma D, Wan X, Civril F, Cleveland DW, Oegema K, Salmon ED, Desai A 2010 Removal of Spindly from microtubule-attached kinetochores controls spindle checkpoint silencing in human cells. Genes Dev. 24, 957–971. (10.1101/gad.1886810) 20439434 PMC2861194

[228] Schmidt JC, Kiyomitsu T, Hori T, Backer CB, Fukagawa T, Cheeseman IM 2010 Aurora B kinase controls the targeting of the Astrin-SKAP complex to bioriented kinetochores. J. Cell Biol. 191, 269–280. (10.1083/jcb.201006129) 20937697 PMC2958477

[229] Dunsch AK, Linnane E, Barr FA, Gruneberg U 2011 The astrin–kinastrin/SKAP complex localizes to microtubule plus ends and facilitates chromosome alignment. J. Cell Biol. 192, 959–968. (10.1083/jcb.201008023) 21402792 PMC3063133

[230] Friese A, Faesen AC, Huis in ‘t Veld PJ, Fischböck J, Prumbaum D, Petrovic A, Raunser S, Herzog F, Musacchio A 2016 Molecular requirements for the inter-subunit interaction and kinetochore recruitment of SKAP and Astrin. Nat. Commun. 7, 11407 (10.1038/ncomms11407) 27095104 PMC4843017

[231] Kern DM, Nicholls PK, Page DC, Cheeseman IM 2016 A mitotic SKAP isoform regulates spindle positioning at astral microtubule plus ends. J. Cell Biol. 213, 315–328. (10.1083/jcb.201510117) 27138257 PMC4862331

[232] Kern DM, Monda JK, Su K-C, Wilson-Kubalek EM, Cheeseman IM 2017 Astrin-SKAP complex reconstitution reveals its kinetochore interaction with microtubule-bound Ndc80. Elife 6, e26866 (10.7554/eLife.26866) 28841134 PMC5602300

[233] Gard DL, Kirschner MW 1987 A microtubule-associated protein from *Xenopus* eggs that specifically promotes assembly at the plus-end. J. Cell Biol. 105, 2203–2215. 2890645 10.1083/jcb.105.5.2203 PMC2114854

[234] Popov AV, Pozniakovsky A, Arnal I, Antony C, Ashford AJ, Kinoshita K, Tournebize R, Hyman AA, Karsenti E 2001 XMAP215 regulates microtubule dynamics through two distinct domains. EMBO J. 20, 397–410. (10.1093/emboj/20.3.397) 11157747 PMC133481

[235] Brouhard GJ, Stear JH, Noetzel TL, Al-Bassam J, Kinoshita K, Harrison SC, Howard J, Hyman AA 2008 XMAP215 is a processive microtubule polymerase. Cell 132, 79–88. (10.1016/j.cell.2007.11.043) 18191222 PMC2311386

[236] Reber SB, Baumgart J, Widlund PO, Pozniakovsky A, Howard J, Hyman AA, Jülicher F 2013 XMAP215 activity sets spindle length by controlling the total mass of spindle microtubules. Nat. Cell Biol. 15, 1116–1122. (10.1038/ncb2834) 23974040

[237] Thawani A, Kadzik RS, Petry S 2018 XMAP215 is a microtubule nucleation factor that functions synergistically with the gamma-tubulin ring complex. Nat. Cell Biol. 20, 575–585. (10.1038/s41556-018-0091-6) 29695792 PMC5926803

[238] Inoue YH, do Carmo Avides M, Shiraki M, Deak P, Yamaguchi M, Nishimoto Y, Matsukage A, Glover DM 2000 Orbit, a novel microtubule-associated protein essential for mitosis in *Drosophila melanogaster*. J. Cell Biol. 149, 153–166. (10.1083/jcb.149.1.153) 10747094 PMC2175100

[239] Lemos CL, Sampaio P, Maiato H, Costa M, Omel'yanchuk LV, Liberal V, Sunkel CE 2000 Mast, a conserved microtubule-associated protein required for bipolar mitotic spindle organization. EMBO J. 19, 3668–3682. (10.1093/emboj/19.14.3668) 10899121 PMC313969

[240] Akhmanova A et al 2001 Clasps are CLIP-115 and -170 associating proteins involved in the regional regulation of microtubule dynamics in motile fibroblasts. Cell 104, 923–935. (10.1016/s0092-8674(01)00288-4) 11290329

[241] Pereira AL et al 2006 Mammalian CLASP1 and CLASP2 cooperate to ensure mitotic fidelity by regulating spindle and kinetochore function. Mol. Biol. Cell 17, 4526–4542. (10.1091/mbc.E06-07-0579) 16914514 PMC1635371

[242] Wordeman L, Mitchison TJ 1995 Identification and partial characterization of mitotic centromere-associated kinesin, a kinesin-related protein that associates with centromeres during mitosis. J. Cell Biol. 128, 95–104. (10.1083/jcb.128.1.95) 7822426 PMC2120339

[243] Walczak CE, Mitchison TJ, Desai A 1996 XKCM1: a Xenopus kinesin-related protein that regulates microtubule dynamics during mitotic spindle assembly. Cell 84, 37–47. (10.1016/s0092-8674(00)80991-5) 8548824

[244] Desai A, Verma S, Mitchison TJ, Walczak CE 1999 Kin I kinesins are microtubule-destabilizing enzymes. Cell 96, 69–78. (10.1016/s0092-8674(00)80960-5) 9989498

[245] Rizk RS, Bohannon KP, Wetzel LA, Powers J, Shaw SL, Walczak CE 2009 MCAK and paclitaxel have differential effects on spindle microtubule organization and dynamics. Mol. Biol. Cell 20, 1639–1651. (10.1091/mbc.e08-09-0985) 19158381 PMC2655246

[246] Domnitz SB, Wagenbach M, Decarreau J, Wordeman L 2012 MCAK activity at microtubule tips regulates spindle microtubule length to promote robust kinetochore attachment. J. Cell Biol. 197, 231–237. (10.1083/jcb.201108147) 22492725 PMC3328376

[247] Maiato H, Gomes AM, Sousa F, Barisic M 2017 Mechanisms of chromosome congression during mitosis. Biology 6, 13 (10.3390/biology6010013) PMC5372006 28218637

[248] Suzuki A, Badger BL, Salmon ED 2015 A quantitative description of Ndc80 complex linkage to human kinetochores. Nat. Commun. 6, 8161 (10.1038/ncomms9161) 26345214 PMC4569735

[249] Roscioli E, Germanova TE, Smith CA, Embacher PA, Erent M, Thompson AI, Burroughs NJ, McAinsh AD 2020 Ensemble-level organization of human kinetochores and evidence for distinct tension and attachment sensors. Cell Rep. 31, 107535 (10.1016/j.celrep.2020.107535) 32348762 PMC7196887

[250] Shelby RD, Hahn KM, Sullivan KF 1996 Dynamic elastic behavior of alpha-satellite DNA domains visualized *in situ* in living human cells. J. Cell Biol. 135, 545–557. (10.1083/jcb.135.3.545) 8909532 PMC2121065

[251] van Hooff JJ, Tromer E, van Wijk LM, Snel B, Kops GJ. 2017 Evolutionary dynamics of the kinetochore network in eukaryotes as revealed by comparative genomics. EMBO Rep. 18, 1559–1571. (10.15252/embr.201744102) 28642229 PMC5579357

[252] Lechner J, Carbon J 1991 A 240 kd multisubunit protein complex, CBF3, is a major component of the budding yeast centromere. Cell 64, 717–725. (10.1016/0092-8674(91)90501-o) 1997204

[253] Cai M, Davis RW 1990 Yeast centromere binding protein CBF1, of the helix-loop-helix protein family, is required for chromosome stability and methionine prototrophy. Cell 61, 437–446. (10.1016/0092-8674(90)90525-j) 2185892

[254] Drinnenberg IA, deYoung D, Henikoff S, Malik HS 2014 Recurrent loss of CenH3 is associated with independent transitions to holocentricity in insects. Elife 3, e03676 (10.7554/eLife.03676) PMC4359364 25247700

[255] Nagpal H, Hori T, Furukawa A, Sugase K, Osakabe A, Kurumizaka H, Fukagawa T 2015 Dynamic changes in CCAN organization through CENP-C during cell-cycle progression. Mol. Biol. Cell 26, 3768–3776. (10.1091/mbc.E15-07-0531) 26354420 PMC4626062

